# From classical machine learning to emerging foundation models: review on multimodal data integration for cancer research

**DOI:** 10.1007/s10462-026-11522-9

**Published:** 2026-03-13

**Authors:** Amgad Muneer, Muhammad Waqas, Maliazurina B. Saad, Eman Showkatian, Rukhmini Bandyopadhyay, Hui Xu, Wentao Li, Joe Y. Chang, Zhongxing Liao, Cara Haymaker, Luisa Solis Soto, Carol C. Wu, Natalie I. Vokes, Xiuning Le, Lauren A. Byers, Don L. Gibbons, John V. Heymach, Jianjun Zhang, Jia Wu

**Affiliations:** 1 Department of Imaging Physics, The University of Texas MD Anderson Cancer Center, Houston, TX 77030, USA; 2 Department of Thoracic/Head and Neck Medical Oncology, The University of Texas MD Anderson Cancer Center, Houston, TX 77030, USA; 3 Department of Thoracic Radiation Oncology, The University of Texas MD Anderson Cancer Center, Houston, TX 77030, USA; 4 Department of Translational Molecular Pathology, The University of Texas MD Anderson Cancer Center, Houston, TX 77030, USA; 5 Department of Thoracic Imaging, The University of Texas MD Anderson Cancer Center, Houston, TX 77030, USA

**Keywords:** Multimodal data fusion, Cancer diagnosis, Prognosis, Machine learning, Deep learning, Foundation models, Biomarker discovery, Artificial intelligence

## Abstract

Cancer research is increasingly driven by the integration of diverse data modalities, spanning from genomics and proteomics to imaging and clinical factors. However, extracting actionable insights from these vast and heterogeneous datasets remains a key challenge. The rise of foundation models (FMs) large deep-learning models pretrained on extensive amounts of data serving as a backbone for a wide range of downstream tasks—offers new avenues for discovering biomarkers, improving diagnosis, and personalizing treatment. This paper presents a comprehensive review of widely adopted integration strategies of multimodal data to assist advance the computational approaches for data-driven discoveries in oncology. We examine emerging trends in machine learning (ML) and deep learning (DL), including methodological frameworks, validation protocols, and open-source resources targeting cancer subtype classification, biomarker discovery, treatment guidance, and outcome prediction. This study also comprehensively covers the shift from traditional ML to FMs for multimodal integration. We present a holistic view of recent FMs advancements and challenges faced during the integration of multi-omics with advanced imaging data. We identify state-of-the-art FMs, publicly available multi-modal repositories, and advanced tools and methods for data integration. We argue that current state-of-the-art integration methods provide the essential groundwork for developing the next generation of large-scale, pre-trained models poised to further revolutionize oncology. To the best of our knowledge, this is the first review to systematically map the transition from conventional ML to advanced FM for multimodal data integration in oncology, while also framing these developments as foundational for the forthcoming era of large-scale AI models in cancer research. The GitHub repo of this project available at https://github.com/WuLabMDA/Medical-Foundation-Models.

## 1 Introduction

Cancer is a leading cause of morbidity and mortality worldwide (Li et al. 2024a), characterized by its complexity, heterogeneity, and adaptability (Berriel Diaz et al. 2024). Its progression is governed by intricate interactions among genetic, epigenetic, proteomic, and metabolic networks, making it one of the most challenging diseases to diagnose, prognosticate, and treat effectively (Yuan et al. 2024). In the era of precision medicine, there is an urgent need for integrative approaches that can unravel these complexities and provide actionable insights into individual tumors’ unique biological and phenotypic characteristics (Zhou et al. 2024b, Cava et al. 2024).

Recent advancements in high-throughput technologies have ushered in the age of multi-omics (Liu et al. 2024b), encompassing genomics (Behrouzian Fard et al. 2024), transcriptomics (Hoang et al. 2024), proteomics (Weaver et al. 2024), metabolomics (Shang et al. 2024), and epigenomics (Arslan et al. 2021). These technologies generate massive datasets that hold the key to understanding cancer at a molecular level, enabling researchers to identify biomarkers (Xiao et al. 2022), elucidate disease mechanisms (Schulte-Sasse et al. 2021), and predict therapy responses (Sammut et al. 2022). Similarly, imaging modalities (Sadeghi et al. 2024) have become indispensable tools in cancer diagnostics (Iranmakani et al. 2020, Mahmood et al. 2021, Abhisheka et al. 2024) and treatment planning (Deantonio et al. 2023, Khalida and Orof 2023). These modalities provide spatial and temporal information about tumor morphology and the surrounding microenvironment (Moody et al. 2021), supplementing the molecular insights derived from omics data (Liu et al. 2024b, Behrouzian Fard et al. 2024, Hoang et al. 2024, Weaver et al. 2024, Shang et al. 2024, Arslan et al. 2021). Building on this, quantitative imaging has emerged as a systematic framework for extracting high-dimensional features from clinical images. Radiomics derives engineered or deep features from routine computed tomography (CT), magnetic resonance imaging (MRI), and positron emission tomography (PET) scans, capturing tumor shape, intensity, and texture (Lambin et al. 2017). Pathomics extends this paradigm to whole-slide histopathology, using patch-level descriptors or deep embeddings to quantify tissue architecture, cellular morphology, and the spatial organization of the tumor microenvironment (Waqas et al. 2025).

However, the integration and analysis of multimodal data present formidable challenges due to the complexity and diversity of the datasets involved (Mohr et al. 2024) (Fig. 1). Molecular data are structured yet high-dimensional and complex (Zhang et al. 2020), whereas imaging data (radiology and histopathology) are high-resolution, spatially rich representations that require specialized feature extraction pipelines to become amenable to statistical modeling (Panayides et al. 2020). In this review, we use “integration” in a broad but precise sense to include (i) vertical integration, where multiple data types (e.g., genomics, transcriptomics, radiomics, pathomics, clinical variables) are linked at the level of the same patient or tumor; (ii) horizontal integration, where cohorts measured with overlapping but non-identical modalities are combined; and (iii) cross-modal alignment of datasets that are only partially overlapping in samples or features, typically via a shared latent representation or alignment objective, in line with recent multi-omics taxonomies. Combining these datasets into unified analytical frameworks requires advanced computational tools capable of capturing intricate relationships across modalities, while handling differing dimensionalities, noise structures, and sampling schemes. Traditional statistical and computational approaches often fall short in handling this complexity, necessitating innovative solutions that can bridge the gap between diverse data types (Bersanelli et al. 2016, Subramanian et al. 2020).

Machine learning (ML), particularly its DL subfield, has revolutionized the integration and analysis of high-dimensional, multimodal cancer datasets (Nakach et al. 2024). ML algorithms, including support vector machines (SVM) and tree-based ensemble methods such as random forests (RF) and gradient-boosted decision trees (e.g., XGBoost), are highly effective in identifying patterns and making predictions from multi-omics data (Libbrecht and Noble 2015, Chierici et al. 2020, El-Nabawy et al. 2021). DL has further extended these capabilities, providing powerful tools to learn hierarchical representations directly from raw or minimally processed data and to capture complex nonlinear relationships; however, this flexibility also makes such models susceptible to overfitting and dataset-specific biases if they are not carefully regularized and rigorously validated (Wang et al. 2024c, Tong et al. 2020). Advanced architectures like convolutional neural networks (CNNs) excel in extracting spatial features from imaging data (Yamashita et al. 2018), recurrent neural networks (RNNs) specialize in sequential data like temporal gene expression (Kordmahalleh et al. 2017, Lugagne et al. 2024), and transformers, with their ability to handle large-scale data and contextual dependencies, achieve outstanding results in cancer diagnosis, prognosis, and therapy response prediction (Liu et al. 2024a, Ali et al. 2023). These AI-driven approaches are bridging the gap between molecular and phenotypic data, offering a deeper understanding of cancer biology.

Recently, the emergence of FMs has changed the landscape of AI. These models are trained on massive datasets, and sophisticated architectures are designed to generalize across a wide range of domain-specific tasks (Moor et al. 2023). In oncology, they demonstrate remarkable capabilities in integrating and interpreting multimodal data, including omics profiles, radiological images, histopathological slides, and clinical records, revealing complex associations between molecular and phenotypic features (Wong et al. 2024, Luo et al. 2023, Cui et al. 2024, Pai et al. 2024, Vorontsov et al. 2024). The evolution of computational oncology is driven by a powerful synergy between advancements in AI (Fig. 2a) and breakthroughs in molecular technology and cancer therapy (Fig. 2b). The data explosion from technologies like Next-Generation Sequencing (NGS) and spatial omics has fueled the development of sophisticated DL models. In turn, these AI advancements now enable personalized therapies and AI-assisted clinical decision support, creating a powerful feedback loop of innovation.

Although previous reviews have examined specific aspects of multimodal integration, most have focused narrowly on particular methodologies, individual cancer types, or single data modalities. As a result, an important gap remains: a comprehensive synthesis that evaluates state-of-the-art ML techniques, emerging foundation models, and their integrated application across diverse cancer contexts. Given the rapid evolution and growing complexity of these technologies, there is an urgent need for a holistic review that not only summarizes recent advances but also clarifies their translational implications and delineates unresolved challenges. In this context, our review makes three main contributions. First, we provide a systematic, cancer-focused overview of multimodal integration methods spanning the full spectrum from classical ML through DL to contemporary FMs, explicitly mapping how model design, data requirements, and validation strategies have evolved over time. Second, we connect methodological innovation to practice by jointly synthesizing algorithmic developments and multimodal integration strategies in relation to concrete clinical endpoints (diagnosis, prognosis, treatment response, and workflow optimization) thereby highlighting where these techniques already influence, or are poised to influence, patient care. Third, informed by close collaboration with clinicians, we articulate a structured set of open challenges and future directions, encompassing data-centric issues, model-centric limitations, and clinical implementation barriers. To this end, these elements position this work as a rigorous, cancer-focused critical appraisal of multimodal integration methods from classical ML to modern FMs, designed to support ongoing research efforts and to help steer the next generation of computational oncology.

## 2 Methods

### 2.1 Evidence acquisition and search strategy

To assemble the evidence base for this review, we performed a structured literature search and screening process focused on multimodal cancer studies that use ML, DL, or FMs. This section details the search strategy, eligibility criteria, and selection workflow for the meta-analysis in Sect. 5.1 using PRISMA methodology (Fig. 3). In parallel, we conducted a complementary narrative scoping of recent cancer-relevant FMs including preprints to construct the FM taxonomy in Sect. 5.4, as many of these models do not yet satisfy the strict inclusion criteria applied to multimodal cancer studies.

A comprehensive literature search was conducted in the National Library of Medicine’s PubMed database (https://pubmed.ncbi.nlm.nih.gov). We considered articles published between 1 January 2019 and 12 November 2024, to capture contemporary ML/DL and FM–based multimodal methods. The core search string combined terms for multimodal/multiomics data, cancer, and AI methods: (multiomics OR “multi-omics” OR “multi omics” OR “integrative omics” OR “integrated omics” OR multimodal) AND (cancer OR tumor OR tumour) AND (“machine learning” OR “deep learning” OR “artificial intelligence” OR “foundation model” OR “neural network”) Reference lists of recent reviews on multi-modal integration and medical FMs were also screened to identify additional eligible studies not captured by the database query. The overall identification and screening process is summarized in Fig. 3.

### 2.2 Eligibility criteria and screening

We applied predefined inclusion and exclusion criteria to focus on original research articles that use ML/DL or FMs for multimodal cancer analysis. The following criteria were applied:

Inclusion Criteria

- Original research articles (not reviews, editorials, or perspectives).
- Human or clinically oriented cancer studies that integrate two or more data modalities (e.g., genomics, transcriptomics, epigenomics, proteomics, histopathology, radomics, clinical variables).
- Use of ML, DL, or FM methods as a central component of the analysis (e.g., for diagnosis, prognosis, risk stratification, response prediction, or treatment planning).
- Full-text available in English.

Exclusion Criteria

- Studies on multi-omics or multimodal integration outside of cancer domain.
- Studies focusing on multi-omics/multimodal data but were not in cancer research were not considered.
- Non-peer-reviewed or low-quality sources (e.g., theses, non-archival workshop abstracts) when they did not provide sufficient methodological detail or validation.
- Non-English publications and duplicate or superseded versions of the same study.

Using PubMed and the search strategy described above, we initially identified 3280 records. Before screening, 514 duplicates, 2037 records flagged as ineligible by PubMed’s automated tools, and 397 records removed for other reasons were excluded, leaving 332 unique records for title–abstract screening. Of these, 193 were excluded as not meeting the multi-modal cancer and ML/FMs focus, and 139 full texts were sought for retrieval. Twenty-eight reports could not be retrieved (e.g., inaccessible for full text or incomplete records), leaving 111 articles for full-text assessment. At this stage, 57 articles were excluded for “below quality and eligibility score” because they lacked essential details on data characteristics, integration strategy, or model validation, or performed only minimal integration without a clear multimodal component. The remaining 54 studies constitute the core evidence base for the meta-analysis discussed in Table 4.

### 2.3 Curation of FMs taxonomy

Because many cancer-relevant FMs are reported as preprints (e.g., arXiv or BiorXiv) and are not yet officially published, they did not satisfy all the above eligibility criteria. To capture this rapidly evolving space, we therefore performed a complementary narrative scoping review for Sect. 5.4. We manually searched PubMed, google scholar, arXiv, and BiorXiv venues using combinations of (“foundation model*” OR “foundation models” OR “pre-trained model*” OR “pretrained model*”) AND (omics OR “multi-omics” OR “multi omics” OR multiomics OR “single-cell” OR “single cell”) AND (multimodal* OR “multi-modal” OR “cross-modal”) AND (“digital pathology” OR “computational pathology” OR “whole slide” OR “whole-slide” OR WSI OR histopatholog* OR CT OR “computed tomography”) AND (cancer OR tumour* OR tumor OR oncology). Candidate models were included in the taxonomy if they: (i) provided a general-purpose pretrained representation (omics, pathology, radiology, or multimodal) relevant to cancer; and (ii) were accompanied by at least one downstream task or application in oncology or have been employed in cancer domains.

## 3 Multi-modal integration methods

The landscape of multimodal integration methods can be systematically understood through a two-tiered classification framework that separates foundational architectural choices from advanced algorithmic strategies. The foundational tier is defined by the timing of integration: early fusion merges raw or processed features at the input level; intermediate fusion combines learned representations within the model architecture; and late fusion ensembles the outputs or decisions from models trained on individual data modalities. Distinct from these architectural blueprints are the advanced, algorithm-based methods that define how integration is computationally performed. This diverse and rapidly evolving category includes hierarchical (Wu et al. 2023), attention-based (Li et al. 2024c), multi-view learning (Blum and Mitchell 1998), graph-based (Ding et al. 2020), and correlation-based strategies (Byron et al. 2021), which are increasingly employed to move beyond simple feature concatenation and model the profound complexity of biological systems (Heo et al. 2021, Shu et al. 2021).

Although many contemporary architectures, particularly large multimodal FMs (Waqas et al. 2024a, Ma et al. 2025, Huang et al. 2024a, Azizi et al. 2023), combine several of these mechanisms within a single network, the two axes we emphasize: (i) the timing of fusion (early/intermediate/late) and (ii) the dominant computational strategy (e.g., attention, graphs, multi-view learning, correlation-based methods) remain useful organizing principles. Rather than prescribing rigid categories, this framework is intended as an extensible lens through which future models, including increasingly unified FMs, can be decomposed and compared. In this review, each modality can correspond to a molecular omics layer (e.g., genomics, transcriptomics, proteomics), an imaging-derived representation (radiomics, pathomics), or clinical variables, all of which must be coherently integrated within a common computational framework. Historically, many of these integration strategies first appeared in classical ML pipelines (e.g., early fusion with SVMs or random forests, late-fusion ensembles of modality-specific classifiers) and were later absorbed into DL architectures and, more recently, FMs-based systems, a trajectory that we analyze in greater depth in Sect. 5.

A second design axis concerns whether data are matched or unmatched. In matched designs, all modalities are measured on the same patient, sample, or cell, enabling direct vertical integration across omics and imaging layers. In unmatched settings, different cohorts, time points, or institutions contribute to non-overlapping subsets of modalities (e.g., one cohort with RNA-seq only and another with histopathology only). Here, integration must rely on alignment via shared features (genes, pathways, cell types), statistical matching, or latent-space coupling rather than straightforward sample-wise concatenation. This distinction is critical: many classical early-fusion pipelines implicitly assume fully matched data, whereas modern deep and foundation-model approaches increasingly target partially matched or entirely unmatched datasets.

Orthogonal to structure is modelling intent. Multimodal models may be trained as singletask predictors (e.g., survival in one cancer type), as multi-task systems optimizing several related endpoints, or as general-purpose pre-trained backbones later adapted via fine-tuning or prompting. The same fusion pattern, for example, intermediate, representation-level fusion with attention can therefore underpin narrowly focused classifiers or broad FMs used as shared encoders. Throughout this section we focus on method classes; modelling intent is revisited explicitly in the FM discussion (Sect. 5.4).

Finally, multimodal integration almost always operates on feature representations rather than raw data streams. For molecular omics, these representations include: (i) handcrafted descriptors (e.g., MACCS/ECFP fingerprints, curated gene signatures, pathway scores); (ii) model-derived or knowledge-enriched features (e.g., 3D structural embeddings from AlphaFold, network-derived centrality or module scores, outputs of prior prognostic models); and (iii) unsupervised or self-supervised embeddings, such as autoencoder bottlenecks, contrastive-learning representations, or latent spaces from FMs. Imaging modalities follow a parallel progression from handcrafted radiomics features texture (Lambin et al. 2017) to CNN or transformer embeddings and joint vision-language representations. The integration strategies surveyed below are largely agnostic to the specific feature type but differ in how effectively they can combine, regularize, and interpret these heterogeneous feature spaces.

### 3.1 Stage-wise fusion methods

Foundational integration strategies are categorized by the time point at which multimodal data are computationally fused. The simplest approach, early fusion, concatenates all feature vectors at the input level before they are fed into a unified model. Conversely, late fusion operates at the output level, where predictions from distinct, modality-specific models are combined for a final decision (Fig. 4). Intermediate fusion offers a compromise, merging learned feature representations at an intermediate layer within a deep learning architecture. This allows initial layers to learn modality-specific features while subsequent layers perform joint analysis. Each strategy offers a different balance between model complexity, modularity, and the ability to capture cross-modal dependencies.

#### 3.1.1 Early (feature-level integration)

Early fusion, or feature-level integration, concatenates features from all modalities into a single representation before learning begins (Leng et al. 2022). If $X^{\left(1\right)},X^{\left(2\right)},\ldots ,X^{\left(m\right)}$ denote the extracted feature matrices from each modality, then the fused input is simply:

(1) $$X_{\text{concat}}=\left[X^{\left(1\right)}|X^{\left(2\right)}|\ldots |X^{\left(m\right)}\right],$$

This integrated feature matrix is then passed to a model to predict the output of interest. Early fusion is conceptually simple and can capture fine-grained cross-modal interactions, but it suffers from two practical challenges: (i) very high dimensionality, which exacerbates overfitting, and (ii) the need for complete data, making the approach brittle when modalities are missing or only partially available (Tong et al. 2020).

#### 3.1.2 Intermediate (representation-level integration)

Intermediate fusion first transforms each modality into a compact latent space before combining them. For each modality, an encoder $g^{\left(i\right)}(\cdot )$ produces an embedding:

(2) $$H^{\left(i\right)}=g^{\left(i\right)}\left(X^{\left(i\right)}\right),$$

These modality-specific embeddings are then merged typically by concatenation, averaging, or an additional fusion module, and passed to a predictive model. Intermediate fusion maintains modality-specific learning while enabling joint representation analysis. However, interpretability can suffer because latent spaces are not always biologically meaningful.

#### 3.1.3 Late (decision-level integration)

Late integration, or decision-level integration, entails training separate models for each modality combines their predictions rather than their features (Leng et al. 2022, Saad et al. 2024). For modality i:

(3) $${\overset{\prime }{Y}}^{\left(i\right)}=f_{\theta }^{\left(i\right)}\left(X^{\left(i\right)}\right),$$

The final prediction $\overset{\prime }{y}$ is obtained by aggregating the individual predictions through an aggregation function ϕ(:).

(4) $$\overset{\prime }{Y}=\phi \left(\left[{\overset{\prime }{Y}}^{\left(1\right)},{\overset{\prime }{Y}}^{\left(2\right)},\ldots ,{\overset{\prime }{Y}}^{\left(i\right)}\right]\right)$$

Late fusion is robust to missing modalities and modular across cohorts but cannot capture feature-level synergies because cross-modal interactions are only considered at the decision level.

Unfortunately, these predefined data fusions are often too rigid, as early fusion requires perfectly aligned data that is sensitive to missing modalities, while late fusion fails to uncover complex, synergistic interactions between the data sources. This static approach often forces a compromise, either losing granular details early on or missing crucial cross-modal correlations by waiting until the very end to integrate insights.

### 3.2 Advanced integration methods

Beyond simple fusion, advanced integration methods employ distinct computational philosophies to harness multimodal data. To reflect known biological hierarchies and dependencies, hierarchical methods construct structured, multi-layered models. For dynamically identifying the most salient features within high-dimensional data, attention-based methods offer a powerful solution, assigning importance weights that also aid interpretability. Multi-view learning addresses the challenge of heterogeneous data by treating each modality as a separate “view,” seeking to learn a shared representation that captures both consensus and complementary patterns. Graph-based methods, including graph neural networks (GNNs), are purpose-built to model the explicit relational structures and interactions inherent in biological networks. Lastly, to reduce dimensionality and discover co-variation patterns, correlation-based methods leverage matrix and tensor factorization or canonical correlation analysis to find shared latent variables.

#### 3.2.1 Hierarchical methods

Hierarchical integration methods leverage the established central dogma of molecular biology as a biologically informed blueprint for multimodal fusion. These approaches impose a directed flow of information across omics layers (genomics, transcriptomics, proteomics, and metabolomics) mirroring cellular regulatory cascades. Each omics dataset is first encoded into a latent representation following the hierarchical order of biological regulation. For the first layer, the latent representation is obtained using a feed-forward neural encoder:

(5) $$H^{\left(1\right)}=f_{1}\left(X^{\left(1\right)}\right)$$

For each downstream biological level ℓ=2,…,L, the representation is conditioned on both its own features and the upstream encoded layer:

(6) $$H^{\left(\ell \right)}=f_{\ell }\left(X^{\left(\ell \right)},{\text{H}}^{\left(\ell -1\right)}\right)$$

To combine information across biological layers, hierarchical importance weights $\alpha^{\left(\ell \right)}$ are computed using a neural scoring function $g_{\text{hier}}(\cdot )$ applied to each layer’s latent representation:

(7) $$\alpha^{\left(\ell \right)}=\frac{\text{exp}\left(g_{\text{hier}}\left(H^{\left(\ell \right)}\right)\right)}{\sum_{k=1}^{L}\text{exp}\left(g_{\text{hier}}\left(H^{\left(k\right)}\right)\right)},\;\text{s.t.}\;\alpha^{\left(\ell \right)}\geq 0,\sum_{\ell =1}^{\text{L}}\alpha^{\left(\ell \right)}=1$$

The hierarchical fusion representation is then obtained by weighing the latent representations according to their biological importance:

(8) $$H_{\text{hier}}=\sum_{\ell =1}^{L}\alpha^{\left(\ell \right)}{\text{H}}^{\left(\ell \right)}$$

Models such as TranPo (Wu et al. 2023) exemplify this strategy by transferring gene-level information to protein-level representations, while Bayesian frameworks like iBAG (Wang et al. 2013) explicitly model regulatory relationships (e.g., copy-number variation → gene expression) within the hierarchical structure. Although biologically intuitive, hierarchical integration methods depend heavily on prior knowledge from pathway or interaction databases, which may limit performance when external annotations are incomplete or inconsistent.

#### 3.2.2 Biological network-based methods

Network-based integration methods project multi-modal data onto known biological interaction networks such as protein-protein interactions (PPIs)(Pan et al. 2022), gene regulatory networks (GRNs)(Shu et al. 2021), or metabolic pathways (Heo et al. 2021) enabling a holistic understanding of complex biological systems. This allows for the tracing of molecular perturbations across biological layers and the identification of influential network modules for biomarker discovery (Zhou et al. 2020, Shi et al. 2024). The core limitation of this approach is its dependence on a ‘ground truth’ network; the resulting insights are therefore only as reliable as the underlying, often incomplete and biased, interaction map. Coupled with the inherent challenge of interpreting complex network topologies, ensuring the biological relevance of purely network-driven findings remains a key concern (Subramanian et al. 2020, Bodein et al. 2022). Each omics dataset $X^{\left(i\right)}$ is first mapped to an initial node feature representation using an encoder:

(9) $$H^{\left(i\right)}=f_{i}\left(X^{\left(i\right)}\right)$$

To integrate regulatory and interaction structure, node features are propagated across the biological network. G=(V,E) using a graph-based message-passing rule:

(10) $$H_{v}^{\prime }=\sigma \left(\sum_{u\in 𝒩(v)}W{\text{H}}_{\text{u}}+\text{b}\right),\forall \;v\in V$$

where 𝒩(v) denotes the neighbors of node v, and σ(⋅) is a nonlinear activation function.

A global network-level representation is then obtained by aggregating node features across the graph:

(11) $$H_{\text{network}}=\text{POOL}\left(\left\{H_{v}^{\prime }\;:\;v\in V\right\}\right)$$

Common pooling operators include mean pooling, sum pooling, or attention-based pooling. The final integrated representation $H_{\text{network}}$ can then be used for prediction or downstream analysis.

#### 3.2.3 Attention-based methods

Attention mechanisms enhance neural networks by allowing the model to focus on the most relevant parts of the input data. In attention-based multi-modal integration, each omics dataset is projected to a fixed dimension latent space using a feed forward neural network $H^{\left(i\right)}=f_{i}\left(X^{i}\right)\in R^{n\times {\text{d}}_{h}}$. Given the latent representation of each omics data, an attention weights $\alpha^{\left(i\right)}$ are computed using neural network $g_{\text{att}}(:)$ as:

(12) $$\alpha^{\left(i\right)}=\frac{\text{exp}\left(g_{\text{att}}\left(H^{\left(i\right)}\right)\right)}{\sum_{j=1}^{m}\text{exp}\left(g_{\text{att}}\left(H^{\left(j\right)}\right)\right)},\;\text{s.t.}\;\alpha_{i}\geq 0,\forall i\in \{1,2,\ldots ,m\},\;\text{and}\;\sum_{i=1}^{m}\alpha_{i}=1$$

The attention weights determine the contribution of each omics modality to the final representation $H_{\text{attention}}$, which is a weighted sum of the individual representations:

(13) $$H_{\text{attention}}=\sum_{i=1}^{i}\alpha^{\left(i\right)}X^{\left(i\right)},$$

The attention-based methods offer adaptive integration while providing improved interpretability of how different omics data contributes to decision making process (Gong et al. 2023).

#### 3.2.4 Multi-view learning methods

Multi-view learning provides a powerful conceptual framework for data integration, treating each omics modality as a distinct ‘view’ or perspective of the same underlying biological system (Rappoport and Shamir 2018, Bauvin 2023). The fundamental characteristic of this integration approach is co-learning (Blum and Mitchell 1998), where the models are trained on separate views, and predictions are combined to maximize the consensus among the models in semi-supervised settings. The reason for its success is discussed in (Nigam and Ghani 2000, Muslea et al. 2006). Multi-view learning is also extended with the kernel learning approach to integrate features from different views. DL-based approaches are also introduced to multi-view integration (Wang et al. 2024c, Chen et al. 2023). Recent studies have focused on semi-supervised learning to capture both global structure and local dependencies across omics data layers (Ge et al. 2023, Ren et al. 2024, Li et al. 2024b, Ye et al. 2023). Furthermore, graphs, autoencoder, and DL-based hybrid approaches also promise to capture local and global dependencies (Liu et al. 2023, Chen et al. 2024e, Zhou et al. 2024a, Si et al. 2024, Cao et al. 2023). Despite their sophistication, these methods are often less effective when significant disagreement exists between views or when one modality is substantially more informative than others, as the drive consensus can suppress critical, unique signals. Furthermore, ensuring that the learned shared space is biologically meaningful, rather than a statistical artifact, remains a significant challenge. Each omics view $X^{\left(i\right)}$ is first encoded into a latent representation using a view-specific encoder:

(14) $$H^{\left(i\right)}=f_{i}\left(X^{\left(i\right)}\right)$$

A shared latent representation Z is then learned by minimizing disagreement across all views:

(15) $$Z=\text{arg}\;\underset{Z}{\text{min}}\sum_{i=1}^{m}L(H^{\left(i\right)},\text{Z})$$

where L(⋅,⋅) is a divergence or consistency loss that measures alignment between a view and the shared space. The final integrated representation is obtained by fusing the aligned view-specific embeddings with learned view weights $\alpha^{\left(i\right)}$:

(16) $$H_{\text{multi - view}}=\sum_{i=1}^{m}\alpha^{\left(i\right)}\;H^{\left(i\right)},\;\text{s.t.}\;\alpha^{\left(i\right)}\geq 0,\sum_{i=1}^{m}\alpha^{\left(i\right)}=1$$

This shared embedding $H_{\text{multi-view}}$ is then used for downstream prediction or clustering tasks.

#### 3.2.5 Graph-based methods

Graph-based methods integrate the topological structure and inherent relationship of omics data by representing multi-modal data $X\in R^{n\times D}$ as a unified graph G=(V,E), where V correspond to biological entities such as genes or proteins, and edges E represent interactions or associations between them (e.g., gene-gene interactions, regulatory links, protein-protein interactions). The connectivity of the graph is encoded by an adjacency matrix A. Graph Neural Net GNNs then employ a layered architecture to learn node embeddings $H^{\left(L\right)}=\left\{h_{1}^{\left(L\right)},h_{2}^{\left(L\right)},\ldots ,h_{n}^{\left(L\right)}\in R^{n\times d^{\prime }}\right.$, where the dimension of learned embeddings is denoted by $d^{\prime }$ and L denotes the layer of GNN. For each GNN layer l the embeddings $H^{\left(l+1\right)}$ are computed using message passing architecture:

(17) $$H^{\left(0\right)}=X,h_{i}^{\left(0\right)}=x_{i},$$

(18) $$\begin{array}{c} h_{i}^{\left(l+1\right)}=\sigma \left(W^{\left(l\right)}h_{i}^{\left(l\right)}+\sum_{j\in 𝒩(i)}\;\varphi^{\left(l\right)}\left(h_{i}^{\left(l\right)},h_{j}^{\left(l\right)},e_{ij}\right)\right), \end{array}$$

where $\varphi^{\left(l\right)}$ is a differentiable function, e.g., weighted sum, or multi-layer perception (MLP), 𝒩(i) is the set of neighbors of a node of node i, $W^{\left(k\right)}$ are learnable weight, and σ is a non-linear activation function. After layer L, the final node embeddings $H^{L}$ can be used to perform prediction.

(19) $$\overset{\prime }{Y}=f_{\theta }\left(H^{L}\right),$$

Apart from single unified graph representations, recent advances have moved to more powerful heterogeneous information networks (HINs)(Ding et al. 2020). This strategy first models each omics dataset as its own graph and then fuses them by defining different types of edges or meta-relations that explicitly annotate the specific biological interactions connecting nodes across these otherwise disparate graphs.

#### 3.2.6 Correlation-based methods

Correlation-based multi-modal data integration methods aim to capture correlations and quantify phenotypic traits, disease progression, and therapeutic responses. Early integration strategies, such as Partial Least Squares Regression (PLSR), focused on discovering latent variables by maximizing covariance between linear projections of omics data (Zhang and Datta 2023). The foundational approach to correlate genomic variants with transcriptomic (Pickrell et al. 2010)., this method links single nucleotide polymorphisms (SNPs) to gene expression across the tissue. The correlated analysis of genomic and transcriptomic can reveal the critical role of tumor evolution and chemotherapy-induced mutagenesis in driving molecular complexity (Byron et al. 2021). Similarly, the Cancer Genome Atlas (TCGA) (Brigham and Hospital 2012) correlated multi-modal data to categorize molecular subtypes, including lung and breast cancer. GTEx Consortium (2017) associates genetic variants with gene expression levels to provide critical insights into tissue-specific regulatory mechanisms. Canonical Correlation Analysis (CCA) is a widely adopted pivotal method for uncovering linear relationships across multi-modal datasets (Rodosthenous et al. 2020). In this context, CCA learns projection vectors that maximize correlation between transformed omics datasets, thereby identifying shared patterns across biological layers and improving interpretability of cross-omics relationships. Given two omics datasets $X^{\left(i\right)}$ and $X^{\left(j\right)}$, CCA seeks linear projections:

(20) $$u=w^{(i)T}X^{\left(i\right)},v=w^{(j)T}X^{\left(j\right)}$$

The objective of CCA is to find the projection vectors $w^{\left(i\right)}$ and $w^{\left(j\right)}$ that maximize the correlation between the projected variables uand v:

(21) $$\underset{w^{\left(i\right)},{\text{w}}^{\left(\text{j}\right)}}{\text{max}}\text{corr}(\text{u},\text{v})=\frac{\text{cov}(\text{u},\text{v})}{\sqrt{\text{var}(\text{u})\;\text{var}(\text{v})}}$$

The resulting shared latent representation capturing cross-modal covariance is expressed as:

(22) $$H_{\text{corr}}=[\text{u},\text{v}]$$

This representation $H_{\text{corr}}$ can then be used for prediction, clustering, or downstream integrative analyses.

Sparse and multiple extensions of canonical correlation analysis such as sparse CCA (SCCA), multiple CCA (MCCA), sparse group CCA (SGCCA), and its DIABLO variant (Data Integration Analysis for Biomarker discovery using Latent variable approaches for ‘Omics studies) were developed to handle large-scale multi-omics by imposing sparsity, using covariance rather than correlation, and enabling feature selection across modalities (Witten and Tibshirani 2009, Du et al. 2023, Huang et al. 2024b, Pusa and Rousu 2024). To better capture non-linear relationships, deep CCA (DCCA) and its multi-view generalizations GCCA and DGCCA, including supervised variants, employ deep networks to learn shared latent representations across omics data (Andrew et al. 2013, Benton et al. 2017, Moon et al. 2022). More recently, advanced DL approaches have shifted multimodal integration toward learning latent cross-modal features directly: self-attention–based transformers uncover subtle dependencies across omics layers for tasks such as survival analysis in lung cancer (Li et al. 2024c), are combined with GNNs for disease classification (Wang et al. 2024d), and are integrated with self-supervised objectives like contrastive learning for broad disease modeling (Zhao et al. 2024); in single-cell profiling, self-attention–driven generative models now set the state of the art for aligning modalities and annotating cell states (Rong et al. 2024).

### 3.3 Comparative appraisal of multimodal integration strategies and recommended use-cases

Across the nine multimodal integration methods discussed above, each offers distinct advantages, limitations, and assumptions that determine its suitability for different biological and clinical tasks (Table 1). Early fusion is simple and captures fine-grained cross-modal interactions but struggles with high dimensionality and missing data, whereas intermediate fusion mitigates these issues through representation learning yet sacrifices some interpretability. Late fusion (Leng et al. 2022, Saad et al. 2024) excels when modalities are incomplete or heterogeneous but cannot model feature-level synergies. Hierarchical models leverage known regulatory order, providing mechanistic interpretability (Wu et al. 2023), but depend heavily on the correctness of biological priors. Graph-based approaches uniquely incorporate interaction networks and pathway structure, making them ideal when relational information is crucial (Ding et al. 2020), although their performance is constrained by the completeness and accuracy of curated biological graphs. Multi-view learning enforces consensus across modalities and is effective when views are complementary but may suppress unique modality-specific signals when disagreement exists (Nigam and Ghani 2000, Muslea et al. 2006). Correlation-based methods (Byron et al. 2021, Pickrell et al. 2010) offer interpretable, statistically grounded insights into shared variation but are limited in capturing nonlinear relationships and require paired samples across modalities. Imaging–omics fusion integrates spatial information from radiology or pathology with molecular data, enabling spatial phenotyping and biomarker localization, but it requires large, high-quality imaging datasets and careful cross-modal alignment. Finally, multimodal FMs provide flexibility, capable of integrating images, omics, and text, but are computationally. Together, these diverse methods illustrate a spectrum of trade-offs between interpretability and flexibility, data requirements and robustness, reliance on biological priors and capacity for discovery, highlighting that method selection must be guided by data characteristics, biological assumptions, and the specific goals of the analysis.

## 4 Tools and applications for multi-modal data integration

### 4.1 Multimodal data bank and cancer applications

The integration of multi-modal data has become a cornerstone in advancing cancer research, mostly in the discovery of biomarkers that are prognostic and predictive of disease outcomes. Leveraging multiple omics layers (Fig. 5) provides a holistic molecular understanding of cancer biology. Each omics type contributes unique insights such as genomics identifies genetic variations linked to disease and treatment response; proteomics explores protein expression dynamics and network configurations; transcriptomics captures RNA-level intermediaries of gene expression; metabolomics uncovers biochemical imbalances associated with tumorigenesis; and interact-omics maps functional protein-protein interactions critical to cellular processes. Emerging studies highlight the power of multi-omics in identifying robust biomarker discovery (Zhang and Datta 2023, Ross et al. 2024, Beechem 2020), cancer subtyping (Wei et al. 2023), therapeutic target identification, drug, and therapy response prediction (Sammut et al. 2022, Wu et al. 2023, Saad et al. 2021), immune checkpoint, and PD-L1 prediction (Mu et al. 2021), recurrence and relapse prediction (Hu et al. 2024, Lan et al. 2024), disease progression modeling (Xu et al. 2021), synthetic lethality detection. On the clinical side, the integrative methods identified in this review are already beginning to influence the translational pipeline by moving beyond purely methodological contributions to address concrete needs in diagnosis, prognosis, treatment selection, and workflow optimization (Upadhyay and Bhandari 2024).

To improve diagnostic accuracy and staging, integrative models are enabling earlier and more accurate cancer diagnosis and staging. For example, the fusion of deep features from whole-slide images (WSIs) and MRIs in (Zhang et al. 2024a) provides a non-invasive method for the early diagnosis of prostate bone metastasis with high Area Under the Curve (AUC: 0.85–0.93). In colorectal cancer, combining pathomics from slides with clinical markers significantly improves staging, with a combined model achieving an AUC of 0.814 on test data. Furthermore, ML analysis of cfDNA liquid biopsy data shows promises for non-invasive diagnosis of pediatric sarcomas with an AUC up to 0.97 (Tan et al. 2024b). Collectively, these studies illustrate how multimodal integration can improve early detection and staging, with direct implications for treatment planning and patient counseling.

For enhancing prognostication and risk stratification, a primary application of these models is the generation of more precise prognostic predictions, allowing for better patient risk stratification. Multi-omics models like Tumor Multi-Modal Pre-trained Network (TMO-Net) (Wang et al. 2024a) and GNNs provide robust survival predictions that outperform single-data-type approaches. Multimodal fusion models are particularly impactful; Pathomic Fusion, which combines histology and genomics, demonstrated a concordance index (C-index) of up to 0.85 for predicting survival outcomes (Chen et al. 2020). Similarly, integrating WSIs with clinical data using multimodal AI led to a hazard ratio of 2.33 for predicting distant metastasis in prostate cancer, directly informing patient prognosis. In principle, such models could support decisions about adjuvant therapy, intensity of surveillance, and enrollment in high-risk clinical trials, although most existing evidence remains retrospective (Ross et al. 2024).

Moreover, we found that some models are pivotal in advancing personalized medicine by predicting responses to specific therapies. A model integrating PET/CT imaging and clinical data can predict PD-L1 expression in Non-Small Cell Lung Cancer (NSCLC) with high accuracy (AUC: 0.82–0.89), which is critical for guiding immunotherapy decisions (Mu et al. 2021). In breast cancer, a “radiopathomics” approach fusing MRI and WSI data successfully predicts chemotherapy response with an AUC between 0.81 and 0.86 (Feng et al. 2022). Another model combining MRI, WSIs, and clinical data achieved a C-index of 0.860 for predicting biochemical recurrence after prostatectomy, helping to identify patients who may require adjuvant therapy (Hu et al. 2024). These examples show that multimodal integration is not only improving risk prediction in abstract terms but also aligning predictions with concrete therapeutic decisions such as immunotherapy eligibility, chemotherapy benefit, and the need for intensified follow-up or additional treatment.

Additionally, AI-driven integration is also streamlining clinical processes. For instance, a 3D ResNeXt model that registers MRI and ultrasound images has been shown to reduce targeting error in prostate cancer biopsies by up to 62%, enhancing the precision of a routine clinical procedure (Guo et al. 2020). Another model provides automated quality assessment for PET/CT scans, ensuring data integrity for clinical use with high inter-rater reliability (Kappa: 0.78–0.80) (Qi et al. 2023). The fusion of endoscopy images and text reports via CNNs and Word2Vec has led to a highly accurate (95.3%) automated screening tool for upper gastrointestinal cancer, demonstrating the potential to improve efficiency in large-scale screening programs (Ding et al. 2020). These workflow-oriented applications primarily impact healthcare efficiency and standardization, reducing manual workload, improving consistency across operators and centers, and potentially shortening time-to-diagnosis.

Several publicly available repositories provide comprehensive multimodal datasets (genomic, transcriptomic, proteomic, epigenomic, metabolomic, pathomics, radiomics and clinical metadata) across a wide range of diseases (Table 2). However, as discussed in Sect. 5.2 and 5.3, realizing their full impact on patient care will require not only methodological innovation but also rigorous evaluation of clinical benefit, reproducibility, and equity.

### 4.2 Conceptual overview of integration approaches and their practical characteristics

To complement the integration framework described in Sect. 3, we summarize the major classes of multimodal fusion strategies using a conceptual, task-oriented perspective (Table 3). This table highlights how each integration category aligns with typical biomedical objectives, interpretability considerations, and input requirements. This structured overview provides readers with practical guidance on selecting an appropriate integration paradigm while maintaining generality across diverse data modalities.

## 5 Results and trends from multimodal cancer fusion studies

Our review and meta-analysis distill the findings from 54 articles that were systematically analyzed to address the objectives of this study. Key characteristics and findings from each study, including methodologies, validation strategies, performance metrics, and data availability, are systematically cataloged to provide a detailed comparative reference (Table 4). In addition to summarizing performance, Table 4 explicitly records source code availability, highlighting substantial heterogeneity to the degree to which current multimodal cancer fusion studies enable independent verification and reuse.

### 5.1 Multimodal data integration in cancer using machine/deep learning

The earliest multimodal integration pipelines in oncology were built around classical ML models applied to hand-crafted features. In these systems, radiomics, pathomics, or engineered multi-omics signatures were first extracted using predefined feature sets, and then concatenated or combined via early, intermediate, or late fusion strategies (Sect. 3). Linear models, SVM, RF, XGBoost, and Cox proportional hazards models were widely used for tasks such as survival prediction, recurrence risk stratification, and treatment response classification (Tan et al. 2024b, Yu et al. 2021, Song 2021). These approaches remain attractive in settings with limited sample size or strong prior knowledge because they are relatively data-efficient, straightforward to train and validate, and often more transparent than very deep networks (Yu et al. 2021).

However, as datasets grew in dimensionality and complexity, particularly when combining multiple omics layers with imaging and clinical variables, the limitations of purely classical models became apparent. Hand-crafted features can miss subtle cross-modal interactions and non-linear structure, and early- or late-fusion schemes built on fixed feature sets often struggle to scale to very high-dimensional spaces without overfitting (Song et al. 2024). These pressures have driven a shift toward deep and hybrid architectures that learn joint representations directly from data, while still using classical ML components (for example, LR, RFs, or Cox models) as prediction heads or baselines (Zhang et al. 2024a, Cao et al. 2020).

In current state-of-the-art multimodal cancer pipelines, deep models provide the representational backbone, with classical methods retaining value as lightweight, interpretable components and competitive baselines. Broadly, two main research directions have emerged: (i) molecular-level multimodal integration, which fuses multiple omics layers to decode cancer biology, and (ii) fusion of tissue-level imaging with genomic and clinical data to construct a holistic view of the patient.

For molecular multimodal integration, several advanced DL models have demonstrated high performance. Variational autoencoders (VAEs) are prominent, with models like TMO-Net using VAEs with a cross-fusion mechanism to create joint embeddings from CNV, mRNA, mutation, and methylation data for prognostic purposes, achieving F1 scores up to 0.92 and a C-index up to 0.80 (Wang et al. 2024a). GNNs have emerged as a powerful tool for modeling the complex interactions inherent in biological data. For instance, GNNs have been applied to RNA-Seq, CNA, and methylation data to predict survival across multiple cancer types with a high C-index, and to integrate protein interaction networks with gene expression to predict the effects of kinase inhibitors with an AUC of approximately 0.8 (Pu et al. 2022). Ensemble methods, such as the DeepProg framework (Poirion et al. 2021), combine multiple deep learning and machine learning models to integrate gene expression, mutation, and methylation data, yielding robust prognosis prediction with C-indices ranging from 0.68 to 0.80 across various TCGA and GEO datasets.

In the multimodal context, the fusion of imaging data (e.g., WSI, MRI, PET/CT) with other data sources is a key area of innovation. CNNs, often based on architectures like ResNet, remain a foundational component. These are frequently combined with traditional ML classifiers, such as in a model that uses both radiomics and deep features from ResNet50 with a LASSO classifier to achieve an AUC of up to 0.93 for early diagnosis of prostate bone metastasis (Zhang et al. 2024a). Similarly, a ResNet-18 model for pathomics combined with logistic regression for clinical markers reached an AUC of 0.907 for colorectal cancer staging (Tan et al. 2024b). More advanced architectures are increasingly prevalent. Vision Transformers (ViT) are being integrated with multi-instance learning to combine WSIs and multi-modal data for colorectal cancer prognosis, achieving an AUC greater than 0.85 (Tsai et al. 2023). Attention mechanisms are central to state-of-the-art fusion models. Pathomic Fusion utilizes a gating-based attention mechanism to integrate histology and genomics, achieving a C-index up to 0.85 for survival prediction (Chen et al. 2020). The SAMMS (Spatial Attention-Based Multimodal Survival) model employs multimodal spatial attention to fuse multimodal and histopathology, reaching a C-index of 0.843 for survival prediction in low-grade glioma (Zhu et al. 2023b).

Furthermore, the field is beginning to leverage large language models (LLMs), with one study integrating LLMs with CNNs/Transformers to fuse clinical text and CT images for bladder cancer survival prediction, demonstrating high data extraction accuracy (87–97%) and an AUC of 0.89. Contrastive learning approaches, such as in the Pathology Language-Image Pre-training (PLIP) model, are creating powerful visual-language FMs for pathology, achieving high F1 scores (up to 0.927) in zero-shot evaluations (Huang et al. 2023).

### 5.2 Reproducibility and proprietary barriers

A striking pattern emerging from Table 4 is that only a subset of high-performing models provide open-source code or fully accessible pipelines, and many rely on institutional or proprietary datasets that cannot be freely shared. This combination of closed implementations and restricted data access poses a significant barrier to reproducibility, independent benchmarking, and fair comparison across methods. In some cases, reported performance cannot be rigorously verified outside the originating center, limiting the community’s ability to assess robustness, detect overfitting, or adapt the models to new populations. For multimodal integration and FMs in particular, where training often requires large-scale, heterogeneous cohorts, these constraints are especially problematic: models trained on proprietary imaging–omics–clinical repositories may encode site-specific biases that are difficult to detect without external replication. More broadly, the lack of standardized reporting on data preprocessing, integration strategies, and cross-site validation further complicates reuse. Going forward, concrete steps such as releasing source code and pretrained weights whenever possible, prioritizing evaluation on public multimodal benchmarks, and adopting transparent reporting checklists (e.g., for data splits, harmonization, and domain shifts) will be crucial to strengthen reproducibility in this field. For settings where data cannot be shared directly, approaches such as federated or privacy-preserving training, shared synthetic benchmarks, and detailed protocol publication offer pragmatic pathways to mitigate the limitations imposed by proprietary datasets and methods (Pati et al. 2022). In this review, we explicitly note where reported gains rely on single-center cohorts, lack external validation, or conflict with findings from related studies, and we qualify our interpretation of such results accordingly in Table 4 and Sect. 5.3. These reproducibility and evidence-quality limitations are further synthesized in Sect. 6.1 as key gaps that must be addressed before many of the surveyed approaches can be considered ready for routine clinical deployment.

### 5.3 Translating technological advances into clinical outcomes

In Table 4, multimodal models are evaluated on a diverse set of endpoints that are directly tied to patient care. Many works focus on survival-related metrics (e.g., overall survival, progression-free survival, biochemical recurrence, or graft-versus-host disease risk), using C-indices (Zhu et al. 2023a) or hazard ratios to quantify prognostic value (Chen et al. 2020, Zhu et al. 2023b). For example, multi-omics survival models such as GGNN (Zhu et al. 2023a), DeepProg (Poirion et al. 2021), SAMMS (Zhu et al. 2023b), and transplant-focused fusion models (Boehm et al. 2022, McCurdy et al. 2022), as well as multimodal colon cancer survival frameworks like PathOmics (Ding et al. 2023) and Pathomic Fusion (Chen et al. 2020). Other studies target early detection or staging, including nodal and distant metastasis prediction and tumor invasiveness from radiology–clinical or pathomics–clinical fusion (Ross et al. 2024, Hu et al. 2024, Tan et al. 2024b, Song 2021). Others target early detection or staging (e.g., nodal metastasis prediction, lesion classification, or tumor invasiveness), treatment response (e.g., chemotherapy efficacy, immunotherapy benefit, kinase inhibitor response (Pu et al. 2022)), or biomarker surrogates such as PD-L1 (Mu et al. 2021) status and microsatellite instability (Cao et al. 2020). A smaller but important subset addresses workflow-centric tasks, including automated PET/CT quality assessment (Qi et al. 2023), multimodal MRI–ultrasound registration for biopsy guidance (Guo et al. 2020), and segmentation of primary and nodal disease from PET/CT or brain MRI using multimodal CNN and U-Net architectures (Fu et al. 2021, Liu et al. 2020, Azad et al. 2024). Taken together, these endpoints illustrate that recent technological advances in multimodal integration and FMs are not operating in a vacuum, but are increasingly aligned with clinically meaningful tasks. For example, models that combine radiology, pathology, and multi-omics to refine risk stratification could enable more granular decisions about adjuvant therapy, surveillance intensity, or trial eligibility (Ross et al. 2024, Hu et al. 2024, Tan et al. 2024b, Chen et al. 2020, Song 2021, Poirion et al. 2021, Zhu et al. 2023b, Zhu et al. 2023a, Boehm et al. 2022). Early metastasis prediction or accurate nodal staging from integrated imaging–clinical models has the potential to reduce unnecessary procedures, prioritize high-risk patients for aggressive treatment, and avoid overtreatment in low-risk groups (Yu et al. 2021, Papp et al. 2021). Similarly, multimodal prediction of treatment response or immune-related biomarkers (such as PD-L1 or MSI) could inform therapy selection, sparing patients from ineffective regimens and associated toxicities (Mu et al. 2021, Feng, 2022, Cao et al. 2020, Pu et al. 2022). Workflow-oriented models, including those for image quality control, registration, and auto-segmentation, primarily target healthcare efficiency by reducing manual workload and variability, shortening time-to-report, and improving consistency across institutions (Guo et al. 2020, Qi et al. 2023, Fu et al. 2021, Liu et al. 2020).

At the same time, most studies in our survey remain retrospective, single- or limited-center analyses, and only a minority are embedded in prospective trials or decision-analytic frameworks. Health-economic endpoints such as cost-effectiveness, resource utilization, or impact on waiting times are rarely quantified. As a result, the link from improved predictive performance to tangible clinical benefit is often inferred rather than directly demonstrated. Bridging this gap will require prospective impact evaluations, integration of multimodal FMs into clinical decision-support tools, and formal assessment of how their use affects survival, toxicity burden, time-to-diagnosis, and healthcare efficiency at the system level. These needs are echoed and further elaborated in the future directions section (Sect. 6.2).

### 5.4 State-of-the-art foundation models

FMs represent a paradigm shift in computational oncology, moving beyond task-specific supervised learning toward architectures that learn generalized representations of complex biological data. By leveraging self-supervision on vast, often unlabeled datasets, these models extract deep, transferable features from individual modalities such as omics, histopathology, and radiology. Building on the classical ML and deep/hybrid architectures discussed in Sect. 5.1 and the integration patterns outlined in Sect. 3, this section has two goals: (i) to summarize the current landscape of medical FMs across modalities, and (ii) to highlight emerging algorithmic innovations and domain-specific design choices that distinguish oncologic FMs from generic vision or language models. Figure 6 provides a high-level taxonomy of FMs in multimodal cancer research, spanning (i) unimodal omics, (ii) pathology, (iii) radiology, and (iv) integrative cross-domain models. In the following, Sect. 5.4.1 offers a model-level survey (complemented by Table 5), while Sect. 5.4.2 focuses on architectural and algorithmic considerations specific to the medical domain.

#### 5.4.1 Taxonomy of cancer foundation models across modalities

FMs are designed to integrate diverse data layers to better characterize cancer heterogeneity, therapeutic vulnerabilities, and disease progression. Table 5 summarizes representative models across four domains—omics-based, pathology-based, radiology-based, and cross-domain multimodal systems—detailing for each the primary objective, training modalities, cohorts, training strategy, and downstream tasks.

##### A. Omics-based and molecular FMs

On the molecular side, several FMs learn high-capacity representations from large-scale multi-omics or single-cell datasets. TMO-Net, for example, adopts a multi-task learning framework including gene mutation, DNA methylation, transcriptomics, and copy number variations (CNVs) to reveal complex patterns in a regularity network (Wang et al. 2024a). This network could be fine-tuned for downstream tasks even with incomplete data modalities. Similarly, scGPT adopts a pretrained transformer for single-cell biology (Cui et al. 2024). The performance of scGPT model is validated for several downstream tasks, such as single-nucleus sequencing for pancreatic ductal adenocarcinoma (PDAC) (Tan et al. 2024a), cell type annotation (Boiarsky et al. 2023), to assess functional characterization of the immune system (Yuan et al. 2024), cross-tissue single-cell annotation (Fischer et al. 2024), generative modeling of omics data (Schuster et al. 2024), and generation of single-cell data (Luo et al. 2024). Nicheformer (Schaar et al. 2024) is designed to analyze single-cell and spatial transcriptomics (ST) data. This model is pretrained on 57 million dissociated and 53 million spatially resolved cells across 73 tissues on cellular reconstruction. The nicheformer is applied in several downstream analysis tasks, such as spatial label prediction, prediction of the effect of unseen single and double perturbations, and single-cell omics (Ahlmann-Eltze et al. 2024). MolFM jointly learns from molecular structures, biomedical texts, and knowledge graphs and is applied to cross-modal retrieval, molecule captioning, and text-based molecule generation tasks (Luo et al. 2023). GenePT (Chen and Zou 2024c) represents genes and cells by leveraging LLM embeddings using text descriptions from the National Center for Biotechnology Information (NCBI). GenePT is applied to gene functionality classification, gene property prediction, gene-gene interaction (GGI), protein-protein interaction (PPI) prediction, and gene perturbation prediction for downstream tasks (Chen and Zou 2024d), while Precious3-GPT adopts a multimodal approach to combine textual, tabular, and knowledge graph representations of biological experiments to study the biological intersections of aging and cancer and evaluate for downstream tasks such as age prediction, drug sensitivity prediction, and gene annotation (Galkin et al. 2024). Collectively, FMs, including TMO-Net (Wang et al. 2024a), P3GPT (Galkin et al. 2024), Path-GPTOmic (Wang et al. 2024e), MolFM (Luo et al. 2023), and Nicheformer (Schaar et al. 2024), have significantly advanced the integration ability of diverse molecular data and offered holistic insights into the complex biological. However, while these models unravel the molecular intricacies of cancer, they lack the spatial and structural information that imaging modalities provide.

##### B. Pathology and radiology FMs

Imaging FMs capture the structural and morphological hallmarks of cancer. Histopathology-focused models such as UNI (Chen et al. 2024a), PLUTO (Juyal et al. 2024), GPFM (Ma et al. 2024), RudolfV (Ma et al. 2024), and CTransPath (Wang et al. 2022) are trained on large collections of WSIs to produce robust slide- or patch-level embeddings. Others, including PRISM (Shaikovski et al. 2024) and CONCH (Lu et al. 2024a), adopt vision–language objectives to align WSIs with clinical reports or captions. Parallel efforts in radiology have yielded dedicated FMs for CT and other imaging modalities. For example, SAM-Med (Huang et al. 2024a), CT-CLIP (Hamamci et al. 2024), and Google’s CT-Foundation model (Yang et al. 2024), which transform volumetric CT data into compact, information-rich embeddings suitable for downstream tasks such as organ segmentation, lesion detection, and cancer risk prediction. These imaging FMs accelerate diagnostics and image-based biomarker discovery by providing rich spatial representations of tumor morphology and microenvironment. Within computational pathology, we note that several domain-specific reviews and toolboxes (e.g., large-scale surveys of AI in digital pathology and open-source frameworks such as TIAToolbox for end-to-end WSI analysis) already cover digital pathology workflows in depth (Pocock et al. 2022); here we focus specifically on their role as FMs within multimodal oncology pipelines.

##### C. Cross-domain and integrative FMs

A smaller but rapidly growing class of models explicitly integrates imaging, molecular, and clinical data within a unified FM. Examples include multimodal architectures such as Path-GPTOmic (Wang et al. 2024e) and P3GPT-like systems, which couple cross-attention or contrastive objectives across WSIs, omics, and textual/clinical inputs. These integrative FMs typically require carefully curated, paired datasets but hold the greatest promise for capturing cross-scale biology and supporting diverse tasks—ranging from survival prediction and treatment response modeling to zero-shot biomarker inference and clinical report generation.

From a comparative standpoint, these model families occupy different positions along the trade-off space between model complexity, data requirements, and performance. Unimodal omics FMs (e.g., scGPT (Cui et al. 2024), Nicheformer (Schaar et al. 2024), MolFM (Luo et al. 2023), GenePT, Precious3-GPT (Galkin et al. 2024)) operate on very large but relatively well-structured datasets and provide strong transferability within the molecular domain, at the cost of substantial pretraining compute. Histopathology and radiology FMs must process giga-pixel WSIs or 3D CT volumes, driving architectural complexity (hierarchical ViTs, MIL-based pipelines) and memory footprint, but yielding highly expressive spatial representations. Integrative multimodal FMs sit at the most demanding end of this spectrum, requiring paired imaging–omics–clinical cohorts and more elaborate fusion modules, yet offering the broadest potential clinical utility (Song et al. 2024). Importantly, in low-data regimes or narrowly defined tasks, simpler classical ML models or shallow DL architectures remain competitive due to lower data requirements, easier calibration, and more straightforward validation.

#### 5.4.2 Algorithmic innovations and domain-specific model design

While Sect. 5.4.1 focuses on what cancer FMs currently exist, an equally important question is how their architectures must be adapted for oncology, beyond simply applying generic vision or language backbones to medical datasets. In practice, contemporary oncologic FMs span a spectrum of modelling intent from task- and domain-specific encoders (e.g., pathology-only or CT-only models optimized for a restricted set of endpoints) to broad, general-purpose representations that are pretrained with generic objectives and then adapted to multiple diagnostic, prognostic, and treatment-related tasks. This intent is layered on top of the integration patterns described in Sect. 3 (e.g., early vs. intermediate vs. late fusion; attention-, graph-, or correlation-based strategies), rather than replacing them. Figure 7 summarizes these design elements across backbone choice, self-supervised pretraining, and downstream adaptation.

Several algorithmic themes are emerging as particularly domain-critical for medical FMs. First, biology-aware architectures increasingly embed prior biological structure—for example, gene-rank tokenizations and pathway-aware attention in omics models, or hierarchical encoders that mirror tissue, organ, and patient-level organization in imaging FMs. These designs move beyond generic spatial or textual encoders toward networks that better reflect biological hierarchies and dependencies. Second, uncertainty quantification and calibration are becoming central requirements for clinical deployment: Bayesian layers, deep ensembles, temperature scaling, and conformal prediction are being coupled to FM backbones to provide calibrated probabilities, uncertainty estimates, and risk strata that can be meaningfully incorporated into clinical decision-making and regulatory assessment (Zhang et al. 2024b).

Third, domain adaptation and robustness are essential because medical data exhibit strong site-, vendor-, and protocol-specific shifts. Domain-aware FMs therefore incorporate components such as conditional normalization layers informed by acquisition metadata, lightweight domain adapters, adversarial domain-invariance losses, and self-normalizing modules (e.g., SeNMo-style architectures (Waqas et al. 2024a)) to maintain performance across institutions and scanners while avoiding spurious correlations. Finally, clinically constrained outputs and interpretability are increasingly built into the design of oncologic FMs. Rather than producing only generic embeddings; these models aim for representations and prediction heads that align with clinically interpretable entities, standardized response criteria, risk scores, staging categories, or biologically meaningful latent factors. As discussed in Sect. 5.1.2.A, this includes not only saliency and attention maps but also structured rationales and chain-of-thought–style explanations (of the kind popularized by modern LLMs such as Gemini, Claude, DeepSeek-R1, and the ChatGPT) that can, in the future, be grounded in specific image regions, laboratory values, or omics features. Together, these algorithmic directions illustrate that medical FMs require domain-optimized model design, not merely medical datasets, to be clinically useful and trustworthy.

### 5.5 Integrating spatial phenotypes with molecular data

The convergence of two powerful technologies enables an unprecedented, spatially resolved view of cancer biology. The first, digital histopathology, transforms tissue slides into giga-pixel WSIs, providing a high-fidelity spatial canvas of cellular architecture and morphology—a gold standard for diagnostics (Omar et al. 2024, Bahadir et al. 2024). The second, spatial omics, overlays this canvas with precise molecular data. Technologies such as Visium HD (Vickovic et al. 2019), digital spatial profiling (DSP) (Beechem 2020, Merritt et al. 2020), and spatial molecular imaging (SMI) (He et al. 2022) now allow for the direct mapping of transcriptomic or proteomic profiles onto tissue structures, creating a one-to-one link between molecular state and histological context (Longo et al. 2021).

This technological synergy has catalyzed two principal paradigms for data integration. The first is in silico molecular inference, where DL models are trained to predict molecular features directly from standard H&E stained WSIs, effectively acting as a computational microscope to bridge histology and genomics when direct spatial data is unavailable. Seminal works like HE2RNA and HE2Gene, often leveraging advanced architectures with self-attention, have demonstrated the potential to infer gene expression profiles from tissue morphology alone (Schmauch et al. 2020, Pronier et al. 2020, Pizurica et al. 2024, Alsaafin et al. 2023, Chen et al. 2024b). The second, more direct paradigm is the fusion of WSIs with concurrently measured spatial omics data. This approach allows researchers to directly correlate molecular activity with histological features like tumor invasion and immune infiltration, offering a more mechanistic view of the tumor microenvironment (Longo et al. 2021, Meroueh et al. 2024, Bai et al. 2024, Pang et al. 2024).

Both paradigms, however, grapple with significant technical hurdles. A primary challenge is the immense data heterogeneity—reconciling giga-pixel image data with sparse, high-dimensional omics matrices. To manage the scale of WSIs, Multiple-Instance Learning (MIL) has emerged as a standard strategy, dividing images into thousands of smaller patches (instances) whose information is then aggregated for joint analysis with omics data (Waqas et al. 2024b, Waqas et al. 2024c). A second major bottleneck is the availability of well-curated, paired (matched) datasets in which histology, spatial omics, and/or radiology are measured on the same tissue specimen or patient. In many practical scenarios, researchers instead rely on unmatched datasets—for example, bulk RNA-seq from one cohort and WSIs from another—where integration must proceed via statistical alignment, domain adaptation, or shared latent spaces rather than one-to-one correspondence. Such unmatched designs greatly expand the usable data pool but introduce additional assumptions and potential biases (e.g., differences in sampling, preparation protocols, or patient mix)(Queiroz et al. 2025), underscoring the particular value of genuinely matched, spatially resolved resources such as TCGA, the Human Tumor Atlas Network (HTAN), STimage-1K4M (Chen et al. 2024g), and Hest-1 K (Jaume et al. 2024) for training and validating integrative models (Longo et al. 2021, Meroueh et al. 2024, Bai et al. 2024, Pang et al. 2024, Chen et al. 2024f).

Finally, the principles of spatial-molecular fusion are now extending from the microscopic scale of pathology to the macroscopic view of radiology. FMs for volumetric imaging, such as those for CT, are pivotal in this expansion (Hamamci et al. 2024, Yang et al. 2024). By transforming massive 3D CT volumes into compact, information-rich embeddings, these models create an “omics-ready” representation of the tumor and its environment. This radically simplifies the technical challenge of integrating radiological phenotypes with genomics or proteomics, complementing the microscopic view provided by histopathology and accelerating the development of holistic biomarker models for precision medicine.

## 6 Open challenges and future directions

### 6.1 Open challenges

The integration of multimodal data, powered by advanced methods, holds immense promise for revolutionizing cancer research, diagnosis, prognosis, and personalized treatment. However, despite significant advancements, major obstacles remain. Crucially, many of these challenges only fully manifest when models are deployed in real-world clinical environments, where technical limitations intersect with regulatory constraints, workflow integration issues, and human factors. These challenges are systematically delineated in the following sections.

#### 6.1.1 Data-centric challenges

The performance, reliability, and ultimate clinical utility of any AI model are inextricably bound to the fidelity, structure, and integrity of the data upon which it is trained. The most sophisticated algorithm cannot overcome the limitations of flawed or poorly harmonized inputs. In the context of multimodal data fusion, many challenges that manifest as algorithmic failures are, in fact, symptoms of deeper, unresolved data-level issues. These foundational gaps, from intrinsic data heterogeneity to the practicalities of missing data and computational scale, represent the primary bottleneck to progress in the field. They arise both within individual modalities and, critically, in their joint representation, where structurally divergent signals must be coherently aligned.

##### A. The conundrum of data heterogeneity and standardization

The central data-centric challenge is reconciling the deep heterogeneity of multimodal datasets. This is not simply concatenating feature vectors, but bridging fundamental differences in data structure, statistics, and semantics. Genomics is often discrete (e.g., somatic mutations), transcriptomics continuous (expression levels), and proteomics semi-quantitative; integrating these with imaging, where information is encoded in spatially correlated pixels/voxels, is far more complex than correcting batch effects within a single modality (Cao and Gao 2022). Naïve fusion of such disparate feature spaces risks substantial information loss.

Compounding this intrinsic heterogeneity is a lack of standardization in acquisition, processing, and annotation. Across and within institutions, changes in protocols, sequencing platforms, imaging scanners, and bioinformatics pipelines introduce technical artifacts and batch effects that powerful but naïve models may misinterpret as biology (Menyhárt and Győrffy 2021, Pan et al. 2022). This “standardization void” limits the usefulness of public datasets and undermines reproducibility and generalizability: models trained on one pipeline often degrade sharply on another, eroding clinician trust, complicating regulatory approval, and contributing to a potential reproducibility crisis (Lin et al. 2025).

These problems are especially acute for imaging-derived features, where radiomics and quantitative imaging have shown strong shifts with vendor, reconstruction kernel, slice thickness, contrast phase, staining protocol, and digitizer settings. Large multimodal FMs pooling CT, MRI, PET, and WSI thus face not only biological variability but also pronounced acquisition- and device-induced shifts; if unmitigated, they may encode scanner identity rather than tumor biology. Addressing this requires a two-pronged strategy that couples’ data-centric standardization with FM design. On the data side, widely adoptable primitives can reduce trivial domain artifacts: systematic capture of scanner and protocol metadata, physical or digital phantoms for cross-site calibration, intensity and stain normalization, and small benchmark calibration sets spanning vendors and institutions (Codella et al. 2024). On the model side, cancer-specific, domain-aware FMs are needed, using shared backbones with lightweight domain adapters, conditional normalization layers parameterized by acquisition metadata, adversarial or contrastive objectives that encourage domain-invariant representations, and self-normalizing modules (e.g., SeNMo-style architectures (Waqas et al. 2024a)) that explicitly regularize across cohorts. Federated learning and multi-site pretraining further allow institutions to contribute data without centralization while still shaping a common representation space (Pati et al. 2022).

##### B. The curse of dimensionality and the signal-to-noise crisis

The integration of multiple omics layers dramatically exacerbates the classic “curse of dimensionality,” or the p>n problem, where the number of features (p) vastly exceeds the number of samples or patients (n). While transcriptomic data alone may comprise tens of thousands of features, combining it with proteomics, genomics, and metabolomics can easily push the feature space into hundreds of thousands or millions. In this regime, models are highly prone to overfitting—capturing spurious cohort-specific correlations rather than generalizable biology—unless strong dimensionality reduction and feature selection are applied.

The challenge, however, is not only dimensionality but also cross-modal amplification of noise. Each modality introduces its own error profile (e.g., sequencing artifacts, antibody cross-reactivity, sample degradation, imaging noise), and their superposition can create complex, non-linear confounders that simple, modality-specific denoising cannot remove. This “signal-to-noise crisis” calls into question the default assumption that “more modalities are always better.” Beyond a certain point, adding a noisy omics layer may inject more confounding variance than useful signal, leading to negative returns on integration. A key future direction is therefore to integrate selectively rather than exhaustively, using meta-learning or data-valuation schemes that estimate the marginal signal-to-noise contribution and cross-modal complementarity of each modality for a given task—identifying, for example, when imaging, spatial transcriptomics, and routine clinical variables combine synergistically and when specific modalities should be downweighted or excluded to preserve robustness (Song et al. 2024).

##### C. Missing modalities

In benchmark datasets, complete multimodal profiles per sample are often assumed; in real clinical settings this is rare. Patients frequently lack specific molecular tests or imaging scans due to cost, contraindications, sample availability, or logistics (Wang et al. 2024a), making partially observed data a pervasive barrier to integration (Zhao et al. 2024). Training models that remain reliable under incomplete data is therefore a core requirement for any clinically viable AI system, not a special case.

A variety of computational strategies have been developed to address this challenge. Probabilistic frameworks, such as Multi-Modal Factor Analysis (MOFA) (Argelaguet et al. 2018) and total Variational Inference (totalVI) (Gayoso et al. 2021), employ Bayesian latent variable models to infer a shared set of underlying biological factors from the available data, gracefully handling instances where entire modalities are missing for certain samples. In the DL domain, techniques like multimodal dropout deliberately omit random modalities during the training process, forcing the model to learn redundant and resilient representations, thereby enhancing its ability to make predictions when faced with incomplete data at inference time (Stahlschmidt et al. 2022). Generative models (VAEs and GANs) can impute missing views by learning non-linear cross-modal relationships from complete cases (Camino et al. 1902). For the unique challenges of sparse and incomplete single-cell data, specialized methods like Joint Sparse Non-negative Matrix Factorization (JSNMF) have been designed to integrate transcriptomic and epigenomic profiles effectively (Ma et al. 2022, Jin et al. 2020), while methods such as WCluster (Duan et al. 2023, Pouryahya et al. 2020) and pipelines like Panpipes (Rich-Griffin et al. 2023) demonstrate how network-based clustering and modular workflows can support subtype discovery and large-scale analysis despite incomplete modalities (Curion et al. 2024).

However, most existing approaches implicitly assume data are “missing at random”. In clinical practice, missingness is often informative: tumour genomics may be absent because a patient is too frail for biopsy, or PET imaging may be missing because care is delivered in a lower-resource setting. In such cases, missingness correlates with health status, socioeconomic context, and patterns of care (Queiroz et al. 2025). Naïve imputation can therefore discard predictive signal and introduce systematic bias (e.g., underestimating risk in frail patients by filling in genomics from healthier cohorts). A key frontier is the development of missingness-aware models that treat the data-availability matrix itself as input, learning from which modalities co-occur or are absent in specific subgroups and how these patterns reshape the complementary information landscape. This shifts the focus from pure imputation to holistic patient modelling, where the process of data acquisition carries as much context as the measurements themselves.

Beyond clinical and logistical factors, patterns of missing data are also shaped by regulatory and technical constraints in multi-institutional cohorts. Privacy and data-protection frameworks such as Health Insurance Portability and Accountability Act (HIPAA) in the United States and the General Data Protection Regulation (GDPR) in Europe frequently require aggressive de-identification (e.g., removal of dates or geographic identifiers) or outright prohibition of centralizing raw imaging, omics, and EHR data (Gomase et al. 2025). To comply, institutions increasingly resort to federated or distributed-learning setups in which data remain local and only model updates are shared, creating a form of “systematic missingness” from the perspective of a global model that never sees the full variable space (Pati et al. 2022). Data-localization policies, heterogeneous consent models, and institutional review board constraints further fragment the landscape, leading to multimodal cohorts that are “artificially incomplete” even when all modalities exist somewhere in the network (Forjaz et al. 2025). Addressing this regulatory layer of missingness will require not only methodological advances such as federated and privacy-preserving learning, secure linkage of distributed datasets, and robust modeling under partial observability, but also harmonized governance frameworks that enable responsible data sharing without compromising patient privacy (Pati et al. 2022).

##### D. The scalability

The sheer scale of modern biomedical data is pushing the limits of conventional computing infrastructure. A single high-resolution whole-slide pathology image can approach a terabyte in size, and integrating such data with multiple omics layers for large patient cohorts generates datasets of peta-scale proportions. The algorithms required to analyze this data often involving high-dimensional statistics, graph analytics, and DL are computationally intensive. Consequently, High-Performance Computing (HPC) has transitioned from a specialized tool to an essential backbone for cutting-edge multi-omics/multimodal research. HPC architectures, which leverage parallel processors, massive, shared memory, and high-speed interconnects, can reduce analysis times from weeks or days to mere hours, making computationally demanding and more accurate integration algorithms feasible for the first time.

However, the reliance on HPC introduces its own set of formidable challenges. First, technical bottlenecks related to data movement, memory limitations, and interoperability between different software frameworks continue to impede the development of seamless and efficient analysis pipelines. Second, and more critically, the high cost and specialized expertise required to build and maintain HPC infrastructure creates a significant barrier to entry. These risks create an “HPC access gap,” where cutting-edge research becomes concentrated in a few elites, well-funded academic centers, thereby stifling innovation at smaller institutions and widening global healthcare and research disparities. Third, the immense power of HPC enables the training of ever larger and more complex “black box” models, which further exacerbates the critical challenge of model interpretability.

This increasing dependence on HPC is fundamentally reshaping the culture of biomedical research. It necessitates a new, deeply integrated tripartite collaborative model, bringing together domain experts (biologists and clinicians), data scientists (ML and statistics experts), and computational scientists (HPC and systems experts). This shift away from the traditional single-investigator lab model is a significant organizational and cultural evolution. It also raises a profound strategic question for the future of the field: should the primary focus be on building ever larger and more powerful supercomputers, or on designing more computationally efficient, “greener” algorithms that can deliver state-of-the-art results on more modest and accessible hardware? Navigating this trade-off between hardware scale and algorithmic intelligence will be critical for ensuring the democratization and long-term sustainability of computational medicine. For multimodal FMs that jointly encode imaging, omics, and clinical data, this trade-off is particularly acute: models that fully exploit cross-modal interactions tend to be among the most computationally demanding, which risks restricting their development, evaluation, and deployment to a small number of well-resourced centers. As illustrated in Fig. 8, representative models across modalities show an approximately exponential growth in parameter count over the last decade, and the bubble sizes provide a relative proxy for per-inference compute and energy requirements based on published FLOP counts and models.

Considered as a whole, these data-centric challenges underscore that multimodal integration is not simply the union of several independent analyses. Instead, it requires carefully engineered data ecosystems in which heterogeneous modalities are collected, standardized, and curated with integration in mind from the outset, so that their complementary strengths, molecular resolution, spatial context, and longitudinal clinical information, can be jointly leveraged rather than competing or confounding one another.

From this perspective, current multimodal FMs do not automatically resolve these data-centric difficulties and, in some settings, may exacerbate them. Large, flexible architecture are particularly prone to encoding site-, scanner-, or pipeline-specific artifacts when training data are only partially harmonized (Tarazona et al. 2020). In practice, a smaller, well-curated unimodal or bi-modal model can be more reliable than a nominally “multimodal” FM trained on heterogeneous, weakly standardized inputs. Thus, the advantages of FMs over classical pipelines are contingent on rigorous data curation and harmonization and cannot be assumed to hold under arbitrary real-world data conditions.

##### E. Data accessibility, governance, and regulatory constraints

Even if technical challenges around heterogeneity and standardization are solved, the development of clinically meaningful cancer FMs is fundamentally constrained by data accessibility and governance. Public, open-source datasets remain indispensable for method development and benchmarking, but they capture only a narrow slice of real-world oncologic practice, often biased toward tertiary centers, specific imaging vendors (Queiroz et al. 2025, Smiley et al. 2025). Truly robust FMs will require access to large-scale, longitudinal, and demographically diverse clinical cohorts, including raw imaging, detailed treatment histories, and high-resolution molecular profiles. Frameworks such as HIPAA, GDPR, and analogous national regulations constrain secondary use, cross-border transfer, and cross-modal linkage; within a single health system, radiology, pathology, genomics, and clinical records may even sit under different consent and governance regimes (Gomase et al. 2025, Forjaz et al. 2025).

If pretraining remains limited to a few permissive centers or regions, cancer FMs will systematically under-represent many populations, tumor types, and health systems, undermining both equity and generalizability. Overcoming this requires treating governance as a design constraint, not an afterthought: technical strategies (federated and secure multi-party learning, differential privacy (Pati et al. 2022)) must be coupled with harmonized data-use agreements and consent frameworks. Only this combination can enable multi-institutional, multimodal training at scale while remaining compatible with legal, ethical, and societal expectations.

#### 6.1.2 Model-centric challenges

Even with access to perfectly harmonized, complete, and high-quality data, the intrinsic characteristics and operational complexities of advanced DL and FMs models present their own set of significant challenges. The focus here shifts from the data to the algorithms themselves. The very properties that make models like deep neural networks so powerful, their ability to learn complex, non-linear relationships in high-dimensional space, also make them opaque, difficult to fuse with disparate data types, and prone to failures in generalization. Bridging the gap between algorithmic capability and clinical reality therefore requires models that are not only accurate, but also interpretable, robust, and operationally manageable. For multimodal FMs in oncology, this entails designing architectures and training schemes that can explicitly model cross-modal interactions (e.g., through cross-attention, hierarchical fusion, or shared latent spaces (Andrew et al. 2013, Benton et al. 2017, Moon et al. 2022)), handle missing or asynchronous modalities, and propagate calibrated uncertainty across all input channels in a way that is compatible with clinical decision-making.

Despite the enthusiasm surrounding multimodal FMs, empirical evidence for their superiority over task-specific DL or classical ML approaches in oncology remains limited. Most published evaluations are retrospective, often single center or few centers, and rely on internal or closely related external test sets. Head-to-head comparisons against strong, carefully tuned conventional baselines are still relatively rare, and truly prospective studies are even scarcer. Consequently, claims of performance gains should be interpreted as promising but preliminary, rather than definitive proof that FMs consistently outperform existing methods across tasks, institutions, and patient populations.

##### A. Model interpretability and explainability

The opacity of many state-of-the-art AI models, specifically transformers or FMs, is arguably the single greatest barrier to their widespread adoption in clinical practice. In high-stake medical decision-making, a prediction, no matter how accurate it is, is insufficient. Clinicians, regulators, and patients require a comprehensible and verifiable reasoning process to establish trust, ensure accountability, and facilitate genuine scientific discovery (Park et al. 2025). This “black box” problem creates a profound trust deficit, as clinicians are justifiably hesitant to base critical patient care decisions on algorithmic outputs they cannot scrutinize or understand.

To address this, explainable AI (XAI) has emerged (Arrieta et al. 2020), but it is crucial to understand the nuanced distinction between two key concepts. Interpretability refers to models that are inherently transparent by design. These models, such as linear regression, decision trees, or rule-based systems, provide a clear relationship between inputs and outputs that is directly understandable by a human user without supplementary tools (Zhu et al. 2025). Explainability, in contrast, typically refers to the application of post-hoc techniques to an already-trained, opaque model to provide an approximation or justification of its behavior. These methods can be model-specific, like Gradient-weighted Class Activation Mapping (Grad-CAM) for convolutional neural networks (Selvaraju et al. 2017), or modelagnostic, like Local Interpretable Model-agnostic Explanations (LIME) (Hassan et al. 2025) and SHapley Additive exPlanations (SHAP) (Ribeiro et al. 2016), which probe the model’s input-output behavior to assign importance scores to features.

While post-hoc explainability methods are a necessary step, they are an imperfect patch rather than a complete solution. These explanations can be fragile, potentially misleading, and may not faithfully reflect the model’s true internal logic. They explain what features the model used but not necessarily why in a way that aligns with human causal reasoning (Sun et al. 2025). A complementary line of work, particularly in large language and multimodal models (e.g., Gemini, OpenAI o-series, Claude, DeepSeek-R1), is the use of self-generated reasoning traces or “chain-of-thought” explanations (Wang et al. 2025). These systems output an explicit, stepwise textual rationale alongside the prediction, bringing model behavior closer to how clinicians articulate reasoning and potentially aid error checking and documentation (Sun et al. 2025). However, such traces are not guaranteed to be faithful—they may serve as post-hoc rationalizations or even hallucinate intermediate steps (Kim et al. 2025). For safety-critical applications, including multimodal FMs in oncology, it will be essential to rigorously evaluate the fidelity of these reasoning traces and to anchor each step in verifiable evidence from the input (e.g., specific image regions, laboratory values, or omics features) before relying on them in clinical workflows (Park et al. 2025).

These challenges are particularly acute for multimodal cancer FMs, whose very strength lies in combining heterogeneous inputs within deep, highly parameterized architectures. Cross-modal attention shared latent spaces, and hierarchical fusion layers further obscure the mapping from raw inputs to predictions, making it difficult to attribute specific decisions to interpretable biological or clinical factors (Song et al. 2024). At present, there is little systematic evidence that existing XAI techniques or reasoning-trace mechanisms provide faithful, regulator-ready explanations for such systems in oncology (Ribeiro et al. 2016, Wang et al. 2025). As a result, interpretability remains a major unresolved barrier that tempers any claims about the immediate clinical readiness of multimodal FMs.

This realization is driving a paradigm shift away from simply “explaining AI” towards creating “collaborative AI”. This involves a deeper integration of clinical expertise throughout the entire model’s lifecycle, a concept known as the “human-in-the-loop” approach. Instead of clinicians being passive recipients of prediction and static explanation, they become active participants in a collaborative reasoning process (Park et al. 2025). The future of clinical AI may lie in systems that do not just provide an answer, but engage in a dialogue, presenting a differential diagnosis, the evidence supporting each possibility, a measure of confidence, and crucially, highlighting instances where its prediction is based on sparse or conflicting data. Such systems reposition AI from an opaque oracle to a cognitive partner that shares part of the reasoning workload while leaving value-laden decisions with clinicians.

##### B. Robustness and generalizability

A model that achieves stellar performance on a curated benchmark dataset may be celebrated in a research paper, but its true value is only realized if it performs reliably in the chaotic and heterogeneous environment of real-world clinical practice. A model’s accuracy on a held-out test set from the same data distribution is a poor proxy for its clinical utility. The most significant challenge in translating AI models from the lab to the clinic is the “generalizability gap”, the sharp drop in performance when a model is deployed on data from new hospitals, different imaging scanners, or diverse patient populations not represented in the original training data.

This failure to generalize often stems from models learning “data leakage” by exploiting spurious correlations or technical artifacts present in the training data. For example, a model might learn to associate a subtle image artifact unique to a particular MRI scanner with a specific cancer subtype, achieving near-perfect classification on the training data for entirely the wrong reasons. When deployed at a new hospital with a different scanner, the model fails completely. This emphasizes the critical importance of both rigorous data harmonization (*as commented in* Sect. 6.1.1) and, crucially, robust validation protocols. Standard k-fold cross-validation is insufficient. True validation requires testing on large, independent, external datasets from multiple institutions, a practice that is becoming the gold standard for assessing real-world performance.

In the end, generalizability cannot be solved by data collection alone; it is computationally and logistically infeasible to collect training data from every possible clinical setting. The solution must also be algorithmic. This points to the vital importance of research into ML techniques for “domain adaptation” and “domain generalization.” These methods aim to build models that are inherently robust to “domain shift”(Stacke et al. 2020); the change in data distribution between training (source domain) and deployment (target domain). These approaches explicitly train a model to be invariant to domain-specific features (e.g., scanner-specific image textures) while focusing only on the domain-invariant features that represent the true underlying biology. This can be achieved through techniques like adversarial training, where a sub-network of the model attempts to predict the data’s domain of origin (e.g., which hospital it came from), while the main predictive model is trained to generate representations that make this task impossible. By forcing the model to discard domain-specific information, it learns a more robust and generalizable representation of the disease. In multimodal FMs, these ideas extend naturally to learning representations that are both cross-site and cross-modal invariant, so that imaging, omics, and clinical text contribute to a stable shared disease representation. The future of reliable clinical AI lies not just in amassing “big data,” but in developing “domain-aware” learning architectures that are built from the ground up to anticipate and master the heterogeneity of the real world.

Notably, increasing model scale does not automatically close this generalizability gap. Large multimodal FMs can still be overfit to subtle, site-specific artifacts or demographic imbalances, and may in fact be more capable of memorizing idiosyncratic patterns in limited datasets. Current reports of improved cross-site robustness are encouraging but remain based largely on retrospective benchmarks with constrained diversity. There is, as yet, insufficient prospective, multi-institutional evidence to conclude that multimodal FMs are inherently more robust or transportable than well-regularized task-specific models, particularly when deployed in resource-limited or data-sparse settings.

##### C. Accountability

The demand for transparent, collaborative AI directly informs the critical issue of accountability. When an AI system contributes to a clinical decision, establishing clear lines of responsibility is not merely a legal or administrative formality; it is a fundamental requirement for ethical practice and patient safety (Wang et al. 2025). Accountability frameworks must address the entire lifecycle of the AI model, from the initial data curation and potential biases embedded within it, to the validation process and post-deployment surveillance {Aldea, 2025 #65}.

In multimodal FMs context, this challenge is magnified. The complexity of the model and the vastness of the data it integrates can diffuse responsibility, making it difficult to pinpoint the source of an erroneous or harmful recommendation. Is the onus on the model developers, the institution that deploys the system, or the clinician who ultimately accepts its guidance? Therefore, establishing robust governance structures is paramount. These structures must define roles, responsibilities, and accountability, ensuring that despite the complexity of the underlying technology, a clear path to redress and continuous improvement exists. Without such a framework, even the most interpretable and collaborative systems will fail to gain the institutional and societal trust necessary for their integration into routine cancer care, leaving their potential to enhance clinical decision-making unrealized.

##### D. Algorithmic Bias, Fairness, and Health Equity

Algorithmic bias and fairness are not abstract concerns but measurable properties of model behavior under distributional shifts (Queiroz et al. 2025, Smiley et al. 2025). In multimodal oncology FMs, several sources of bias frequently coexist: sampling bias (over-representation of patients from high-income regions or tertiary centers), measurement bias (systematic differences in scanners, staining protocols, or sequencing platforms), label bias (subjective or inconsistently applied clinical labels), and representation bias (under-representation of specific demographic or clinical subgroups in the feature space) (Queiroz et al. 2025). Multimodal integration introduces an additional, less discussed dimension: modality-access bias. Advanced imaging (e.g., PET/CT, whole-slide imaging) and multi-omics profiling are more commonly available in well-resourced settings, such that the “full” multimodal stack is inherently skewed toward already advantaged patients.

These biases can lead to heterogeneous error profiles across subgroups, even when global metrics appear satisfactory (Zhu et al. 2025). For instance, a survival FM trained on imaging–omics–clinical data from a comprehensive cancer center may achieve strong overall C-index yet substantially underperform in patients from community hospitals where molecular profiling is sparse and imaging protocols differ. In a multimodal setting, the interaction between modalities can also amplify bias: if certain combinations of modalities (e.g., WSI + RNA-seq + detailed clinical text) are predominantly observed in younger, insured, or majority-ethnicity patients, the learned latent space may devote the highest capacity to these strata, while systematically degrading performance on under-represented groups or reduced-modality pathways.

From a technical standpoint, fairness assessment in multimodal FMs should therefore go beyond a single aggregate AUC or C-index. At minimum, models should be evaluated using subgroup-stratified metrics (e.g., AUC, calibration error, and Brier score stratified by sex, age bands, race/ethnicity where available, site, and stage), and, where appropriate, formal fairness criteria such as equal opportunity (similar sensitivity across groups for positive cases), equalized odds (similar sensitivity and specificity), or worst-group risk (distributionally robust evaluation). In multimodal contexts, it is also informative to report performance as a function of modality availability patterns (e.g., imaging-only vs. imaging+omics vs. omics+clinical) to detect systematic failures when certain modalities are missing or degraded—an issue tightly coupled to real-world inequities in diagnostic access. Mitigation strategies likewise need to be adapted to the multimodal setting. At the data level, approaches such as reweighting, stratified sampling, or targeted enrichment of under-represented subgroups can reduce imbalance, while care must be taken not to overfit to small sub-cohorts (Fig. 9). At the model level, fairness-aware objectives (e.g., adding penalty terms for subgroup performance gaps), adversarial debiasing (training latent representations that obfuscate protected attributes while preserving predictive signal), and distributionally robust optimization (explicitly optimizing performance under worst-case subpopulation shifts) provide principled ways to trade off average accuracy against equity. Importantly, these techniques should be evaluated transparently, reporting not only gains in fairness but also any associated changes in overall performance and uncertainty (Zhang et al. 2024b).

Embedding multimodal FMs within a responsible AI framework requires operational components in addition to algorithms. Detailed documentation of data provenance, cohort composition, and inclusion/exclusion criteria (e.g., through model cards and dataset “data-sheets”), explicit logging of how each modality contributes to the decision, and governance structures for periodic fairness audits are all essential (Queiroz et al. 2025). Prospective studies should incorporate equity-focused endpoints such as differential impact on treatment allocation, trial eligibility, or time-to-diagnosis across subgroups, rather than treating fairness solely as an offline metric. Finally, bias and fairness analyses should be integrated into regulatory review and post-market surveillance, with monitoring pipelines that flag performance drift or emergent disparities as practice patterns, populations, and modality availability evolve. Only under such a responsible AI regime can multimodal FMs realistically contribute to narrowing, rather than widening, existing inequities in cancer care.

##### E. Fusing imaging with molecular data

The fusion of spatial data from medical imaging with non-spatial data from omics represents a unique and formidable frontier in multimodal integration. The challenge is not merely computational but deeply conceptual, requiring the invention of novel frameworks that can bridge the vast semantic and structural gap between pixels and molecules. A primary technical hurdle is the co-registration and alignment of data acquired at vastly different scales and resolutions. For example, accurately mapping a 50-micron spot from a ST array to the precise morphological features within a giga-pixel whole-slide histopathology image is a non-trivial task where even minor misalignments can lead to profoundly incorrect biological conclusions (Waqas et al. 2025).

This challenge is exemplified by the integration of ST with histopathology. While ST provides unprecedented insight into the spatial organization of gene expression within a tissue, its utility is hampered by persistent technical issues, most notably the accuracy of cell segmentation. Inaccuracies in delineating cell boundaries can create artifacts that make cells appear more transcriptionally similar to their immediate neighbors than they truly are, potentially obscuring genuine cell-cell interaction signals and confounding biological interpretation. The goal of this integration, known as “pathogenomics”, is similar to radiogenomics, which develop models that can predict molecular states, such as the presence of a specific gene mutation or the activity of a signaling pathway, directly from routine, non-invasive clinical images. Achieving this requires AI architectures, such as multi-branch neural networks, capable of learning a shared latent representation where features from these incongruent modalities can be meaningfully compared and fused. In practice, this argues for models that not only align representations across modalities, but also explicitly model cross-modal attention, enforce consistency between image-derived and omics-derived predictions, and surface discordances that may signal biological novelty or technical failure. Figure 10 illustrates how spatial imaging and omics data can be jointly processed through a unified multimodal model to yield clinically actionable pathogenomic insights.

##### F. Clinical workflow integration, regulation, and real-world deployment

Even the most sophisticated multimodal FMs (Huang et al. 2024a, Chen et al. 2024a, Juyal et al. 2024, Ma et al. 2024, Wang et al. 2022, Shaikovski et al. 2024, Lu et al. 2024a, Hamamci et al. 2024, Yang et al. 2024) remains purely academic until it can be safely, reliably, and efficiently embedded within routine clinical workflows. Practical implementation poses a distinct set of challenges that go beyond algorithms and data {Aldea, 2025 #65}. At the workflow level, AI systems must integrate with existing hospital infrastructure (e.g., EHR, PACS, laboratory information systems) without adding unacceptable latency or cognitive burden to already overextended clinicians. Multimodal models that require simultaneous access to imaging, pathology, and multi-omics data must respect the asynchronous and often fragmented nature of real-world clinical pathways, where different modalities become available at different time points and sometimes not at all.

From a regulatory perspective, multimodal FMs deployed for diagnosis, prognosis, or treatment recommendation are typically classified as software as a medical device (SaMD). This entails rigorous documentation of training data provenance, performance characteristics across subgroups, model update policies, and post-market surveillance procedures. For systems that continuously learn from new data or adapt to local practice patterns, regulators increasingly require explicit mechanisms for change control and re-validation, as well as clearly defined processes for monitoring model drift and clinical impact over time. Experience with the first wave of FDA-cleared AI/ML-based medical devices has already demonstrated that regulatory approval is a necessary but far from sufficient condition for sustained clinical value: tools that perform impressively under carefully controlled conditions may show attenuated benefit, performance drift, or inequitable error patterns when exposed to the full heterogeneity of real-world practice (Lin et al. 2025). These lessons are specifically salient for multimodal FMs, whose complexity and dependence on multiple data streams amplify both the potential for impact and the risks associated with unmonitored deployment. Beyond regulatory clearance, successful translation also depends on alignment with reimbursement pathways (e.g., procedure codes, value-based care metrics) and clear medico-legal guidance on how algorithmic recommendations should be incorporated into clinical responsibility frameworks.

Finally, successful deployment demands attention to human factors and organizational readiness {Aldea, 2025 #65}. In practice, many clinicians remain reluctant to rely on complex AI or FM-based systems, which makes it essential that they understand not only what the model predicts, but also under which conditions its recommendations are trustworthy or should be actively discounted, specifically for multimodal FMs whose outputs derive from intricate, non-intuitive interactions between imaging, molecular, and clinical signals. Implementation of science studies, prospective trials, and user-centered design are therefore essential complements to technical development and to the early proof-of-concept studies that currently dominate multimodal FM literature. Without such efforts, many multimodal FMs are likely to remain confined to isolated pilot projects and retrospective demonstrations rather than achieving the scalable, equitable impact on cancer care that they promise. Prospective impact evaluations, pragmatic trials, and real-world evidence studies that embed multimodal FMs into decision-support tools and quantify changes in survival, toxicity burden, time-to-diagnosis, clinician workload, and workflow efficiency will be key to closing the gap between technical performance and genuine clinical benefit.

For multimodal cancer FMs, this evidentiary gap is currently substantial. Most published models report technical metrics on retrospective cohorts, but few studies have demonstrated improvements in hard clinical endpoints, within prospective or pragmatic trial settings. Regulatory approvals specifically targeting multimodal FMs in oncology are essentially absent, and there is limited data on cost-effectiveness, workflow impact, or medico-legal acceptability at scale. Accordingly, the present state of the field should be viewed as an early translational phase: FMs are powerful research tools with clear potential, but their clinical utility and safety remain to be established through rigorous, longitudinal evaluation.

### 6.2 Future directions

While the field has made remarkable progress, the next phase of multimodal data integration in oncology will be driven by four priorities: (i) developing unified and dynamic foundation models, (ii) achieving mechanistically grounded interpretability, (iii) addressing data scarcity through principled generative modeling, (iv) implementing continual learning paradigms that keep models aligned with evolving clinical practice, and (v) reconciling global generalization with local, site-specific adaptation in clinical deployment. Below, we outline concrete directions that are technically feasible in the short-to-medium term and necessary for long-term clinical impact. The priorities outlined above are therefore aspirational and conditional: their eventual impact will depend on whether the community can address the data, model, and governance challenges, and on whether future studies demonstrate clear, reproducible benefits over established analytic pipelines in real-world settings.

#### A. Beyond specialization: towards unified and dynamic foundation models

Current medical imaging and multimodal FMs including UNI (Chen et al. 2024a), PLUTO (Juyal et al. 2024), GPFM (Ma et al. 2024), RudolfV (Dippel et al. 2024), CTransPath (Wang et al. 2022), PRISM (Shaikovski et al. 2024), CONCH (Lu et al. 2024a), REMEDIS (Azizi et al. 2023), TITAN (Ding et al. 2024), TMO-Net (Wang et al. 2024a), P3GPT (Galkin et al. 2024), Path-GPTOmic (Wang et al. 2024e), MolFM (Luo et al. 2023), Nicheformer (Schaar et al. 2024), SAM (Huang et al. 2024a), CT-CLIP (Hamamci et al. 2024) and CT-foundation google (Yang et al. 2024) demonstrate substantial potential but remain largely specialized, typically constrained to a single modality or a narrow set of predefined combinations. A key next step is the development of unified multimodal FMs that can jointly encode imaging, multi-omics, and clinical data within a shared representational space. In the near term (3–5 years), actionable directions include:

- Designing cross-modal transformer architectures that support plug-and-play encoders for imaging, omics, and clinical text, with cross-attention layers explicitly modeling interactions between modalities.
- Establishing public benchmarks and challenge datasets where imaging is systematically linked to multi-omics and longitudinal clinical outcomes, enabling reproducible comparison of multimodal FMs.

In the longer term, models should move beyond static, single-timepoint representations toward spatio-temporal FMs that capture disease evolution:

- Spatially aware pathway modeling, where enriched molecular pathways are mapped onto tumor habitats, immune niches, or invasive fronts on histopathology or radiology.
- Temporally aware dynamic modeling, integrating serial imaging, treatment timelines, and temporal omics to predict resistance, relapse, and immune responses, potentially via temporal graph networks or dynamic Bayesian models will be essential to proactively inform treatment strategies rather than retroactively explaining failure.

#### B. The imperative for mechanistic interpretability

For multimodal FMs to transition from promising prototypes to dependable clinical tools, interpretability must evolve from post-hoc correlation maps to mechanistically informative explanations. A concrete avenue is to use spatial omics and single-cell data as biological “ground truth” to verify whether image regions or latent factors prioritized by the model are enriched for specific cell states, pathways, or microenvironmental niches. Our recommendations for further research include:

- Developing joint visualization frameworks that align model attention maps with spatial omics readouts, allowing clinicians and biologists to interrogate whether predicted signatures are biologically plausible.
- Designing interpretable embedding spaces in which axes and clusters are explicitly annotated with known pathways, immune phenotypes, or stromal states, enabling mechanistic hypotheses to be generated rather than just black-box risk scores.
- Conducting prospective, clinician-in-the-loop studies that evaluate how multimodal explanations influence diagnostic confidence and treatment decisions, thereby quantifying the clinical utility of interpretability rather than treating it as a purely technical metric.

These efforts are immediately feasible with existing datasets and will be essential for regulatory acceptance and clinician trust.

#### C. Generative models as a solution to data scarcity

The scarcity of large, harmonized multimodal datasets remains a major bottleneck. Advanced generative models, progressing from early GANs to guided diffusion and flow-matching models, offer a principled route to augmenting and stress-testing multimodal pipelines, especially for rare cancer subtypes and under-represented populations. The near-term research directions include:

- Fine-tuning large, pretrained generative FMs to synthesize paired imaging–omics–clinical samples under explicit biological constraints (e.g., preserving known pathway structure or mutational patterns).
- Developing task-specific synthetic data protocols, where generative models are used not to replace real data but to: (i) rebalance class distributions, (ii) simulate edge cases (e.g., rare mutations), and (iii) probe model robustness to control distribution shifts.
- Establishing standardized evaluation frameworks for synthetic data that go beyond low-level similarity metrics, focusing instead on functional validation. For example, demonstrating that models trained with synthetic augmentation generalize better on independent real-world cohorts or can rediscover known clinicogenomic associations.

In the medium term, combining generative models with federated or privacy-preserving learning could enable multi-institutional multimodal FM training without centralizing sensitive data, offering a pragmatic path towards larger and more diverse datasets (Pati et al. 2022, Lu et al. 2024a, Ogier Du et al. 2023).

#### D. Global generalization versus local adaptation

Building on the challenges outlined in Sect. 6.1, 6.2 and the FM taxonomy in Sect. 5.4, this review considers large, multimodal cancer FMs trained across institutions and countries as a promising research direction, rather than a settled solution, for future oncology AI. However, real-world deployment will rarely be truly “universal.” In practice, most hospitals operate within comparatively homogeneous environments: a limited set of scanner vendors and protocols in radiology, locally standardized staining and workflows in pathology, and institution-specific patient demographics and referral patterns. Domain shift between centers has been repeatedly shown to degrade model performance in imaging AI (Upadhyay and Bhandari 2024) even for relatively simple chest X-ray and CT tasks, so models validated at one site often underperform when deployed “as is” at another. Critically, this hierarchical view of global backbones and local adapters remains a working hypothesis. It will require formal testing in prospective, multi-site studies to determine whether such FM-based strategies consistently outperform simpler, locally optimized models, and under which deployment conditions, their additional complexity is justified.

A pragmatic view is therefore to treat cancer FMs as backbones rather than monolithic end-products. Pretraining should exploit as much cross-site diversity as governance allows us to learn broad, transferable representations, whereas deployment will typically require local specialization: lightweight fine-tuning or adapter modules on a hospital’s own data, site-specific normalization and calibration layers, and, where direct data pooling is impossible, federated or multi-site fine-tuning protocols (Pati et al. 2022). In this framing, the key design question is not “global or local?” but how best to combine global robustness (learned from heterogeneous, multi-institutional cohorts) with local optimization (to match specific equipment, workflows, and populations) for tasks such as image-based diagnosis, genomic interpretation, and prognostic modelling.

Practically, this points toward hierarchies of models: a shared, multimodal cancer FM pretrained across centers; institution- or region-level variants adapted via domain adaptation and site-specific adapters; and, in some cases, task-specific heads tuned to specific clinical endpoints. Such a tiered strategy acknowledges regulatory and operational realities while preserving the scientific value of globally pretrained backbones.

#### E. Continual learning

A crucial next step for cancer FMs is to move beyond one-off training and periodic offline updates toward continual learning frameworks that can absorb new data, tasks, and clinical knowledge without catastrophic forgetting (Yang, 2025b). In practice, this means treating the FM as a living backbone that is incrementally updated as institutions acquire new imaging protocols (Perkonigg et al. 2021), therapies, and patient populations, while preserving performance on prior cohorts. Techniques such as regularization-based approaches (e.g., elastic weight consolidation), replay or rehearsal of curated reference cohorts, and modular adapter layers offer complementary strategies to balance plasticity and stability in this setting. For oncology, continual learning is particularly important for modelling shifting standards of care (e.g., new immunotherapies), evolving resistance patterns, and changing screening practices, so that deployed FMs remain calibrated, clinically aligned, and empirically valid over time rather than becoming static “snapshots” of past practice.

## 7 Conclusions

To conclude, the integration of multimodal data with advanced AI holds an undeniable and revolutionary promise for oncology. Yet, the path from computational proof-of-concept to meaningful clinical impact is fraught with profound and deeply interconnected challenges. This review has systematically deconstructed these hurdles, revealing that they are not isolated issues to be solved in sequence, but a complex, interdependent system requiring a holistic and synergistic strategy. Our analysis reveals that the advancement of AI models is fundamentally reshaping the analysis of multi-modal and multimodal data. These state-of-the-art models excel at creating unified data representations, uncovering complex biological interactions, and delivering robust, scalable solutions for critical tasks like cancer subtyping, biomarker discovery, and survival prediction.

Clinically, these technological advancements are directly enhancing the translational pipeline, moving precision oncology from an aspirational goal to a clinical reality in a few years. The integrative methods reviewed here are yielding tangible improvements in early and non-invasive diagnostics, enabling more accurate prognostication, and personalizing therapeutic strategies by predicting patient response to specific treatments.

Despite this rapid progress, significant hurdles remain in the path to routine clinical deployment. The field must urgently address the need for standardized, multi-institutional validation protocols to ensure model robustness and generalizability, overcome challenges related to data harmonization, and enhance model interpretability to build clinical trust. Future efforts must be intensely focused on bridging the gap between computational innovation and real-world clinical utility. This will require fostering deep collaboration between data scientists and clinicians, promoting the development of accessible open-source tools, and establishing clear regulatory pathways to ensure that these transformative technologies can be safely and effectively integrated into patient care, ultimately realizing the promise of data-driven, personalized oncology.

## Figures and Tables

**Fig. 1:**
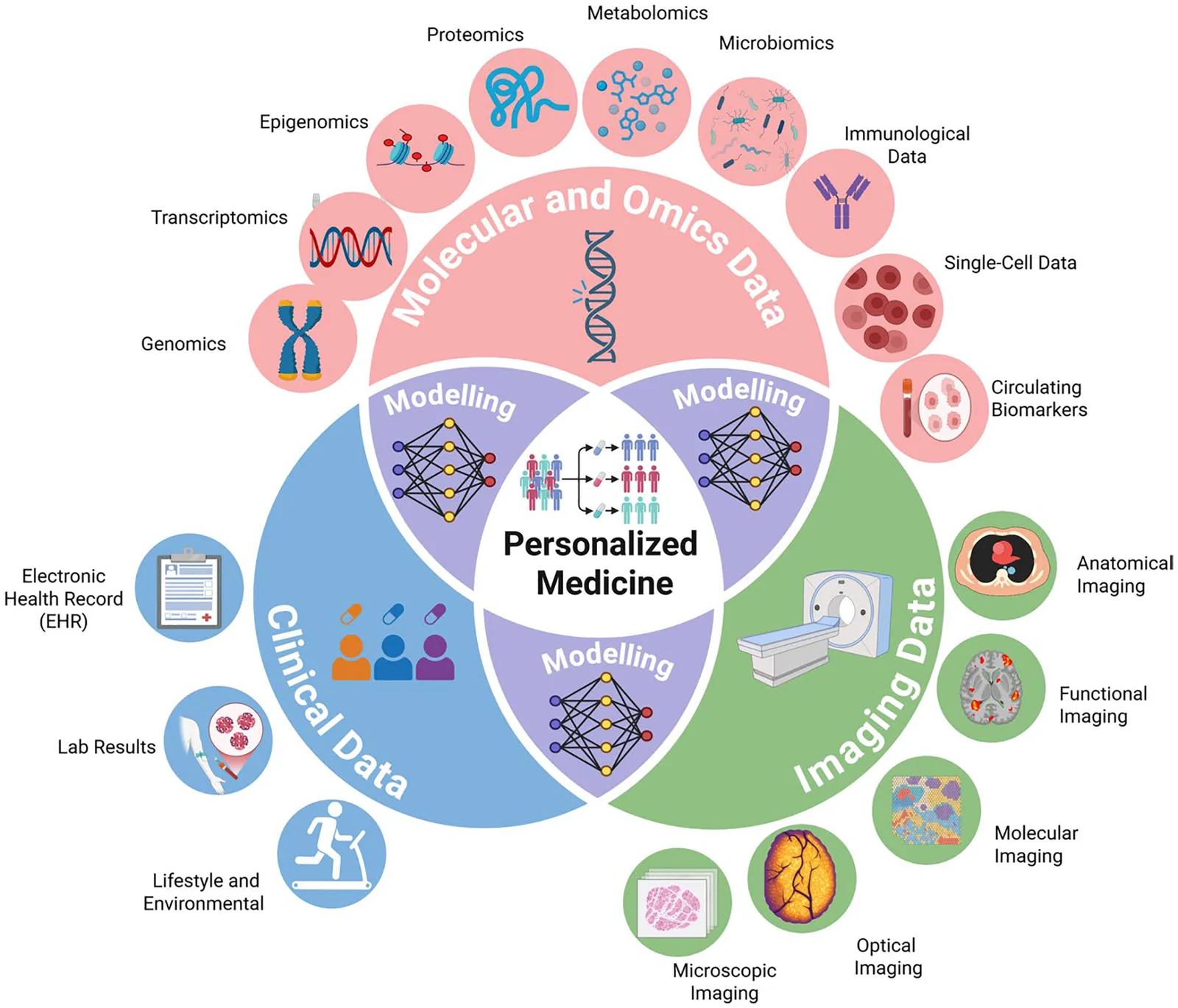
Multi-modal data integration towards personalized medicine

**Fig. 2:**
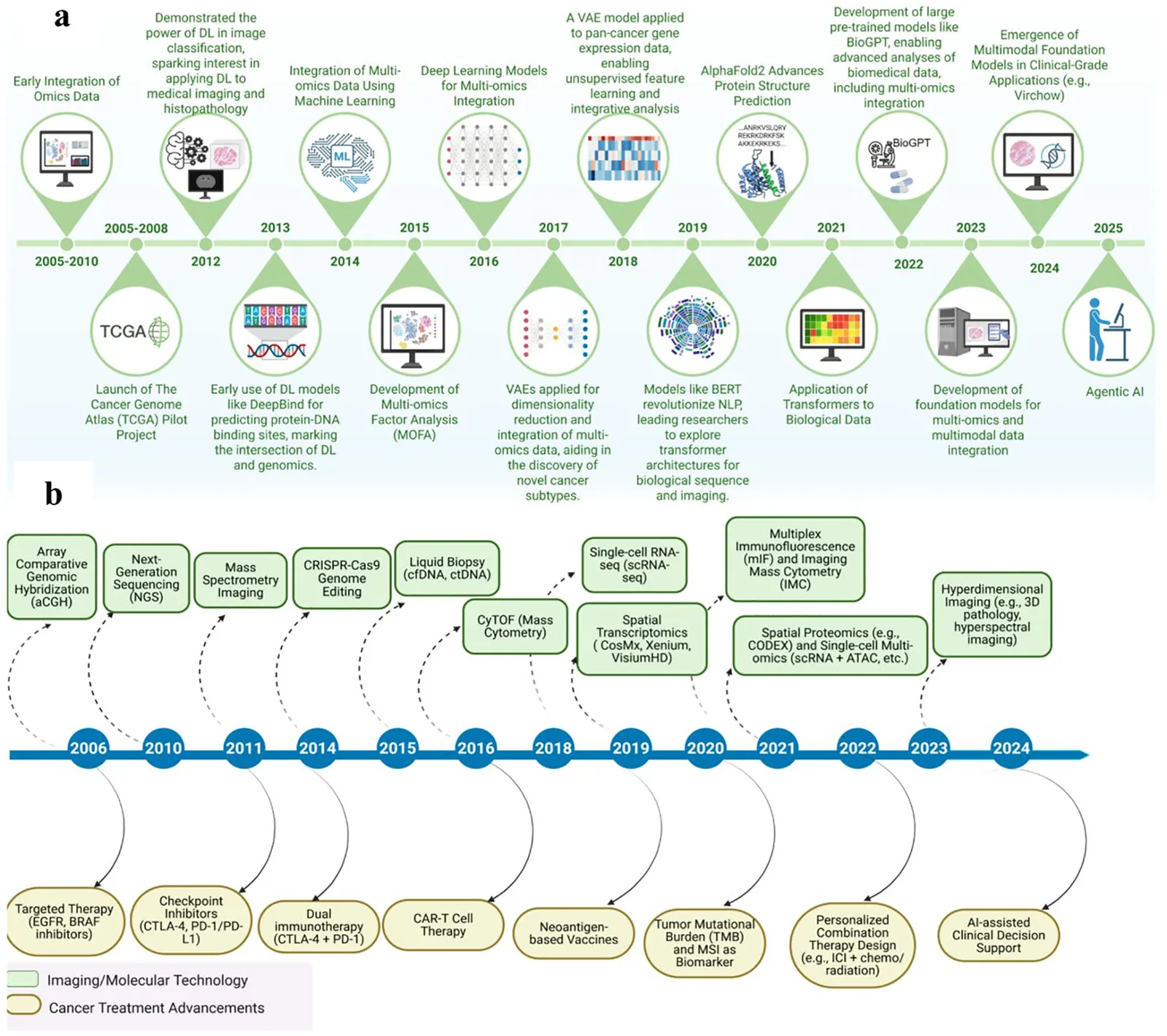
Timeline of (**a**) DL Advancements in Cancer Research through Multimodal Approaches; **b** Imaging/molecular technology and cancer treatment

**Fig. 3:**
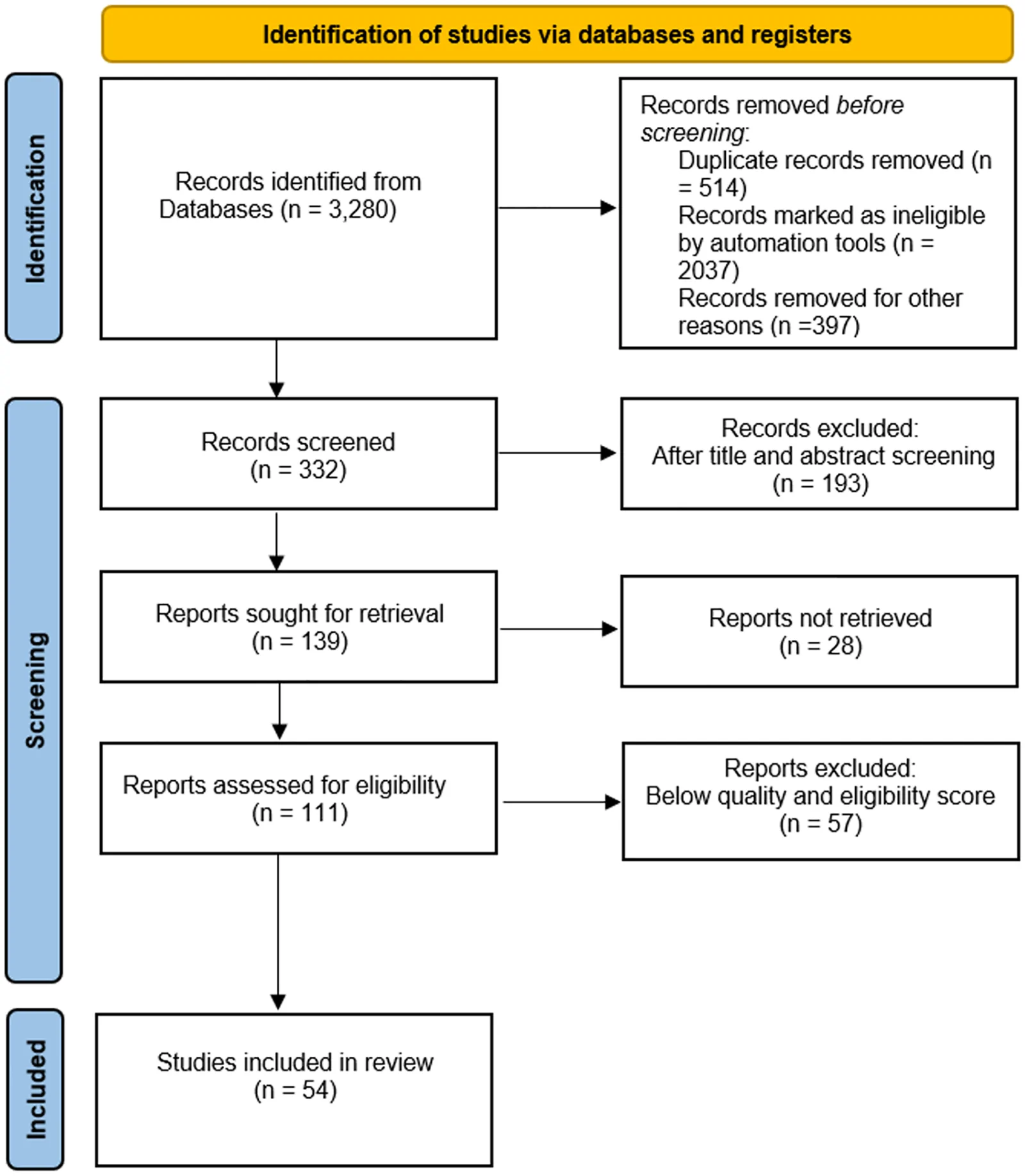
PRISMA methodology for study selection

**Fig. 4:**
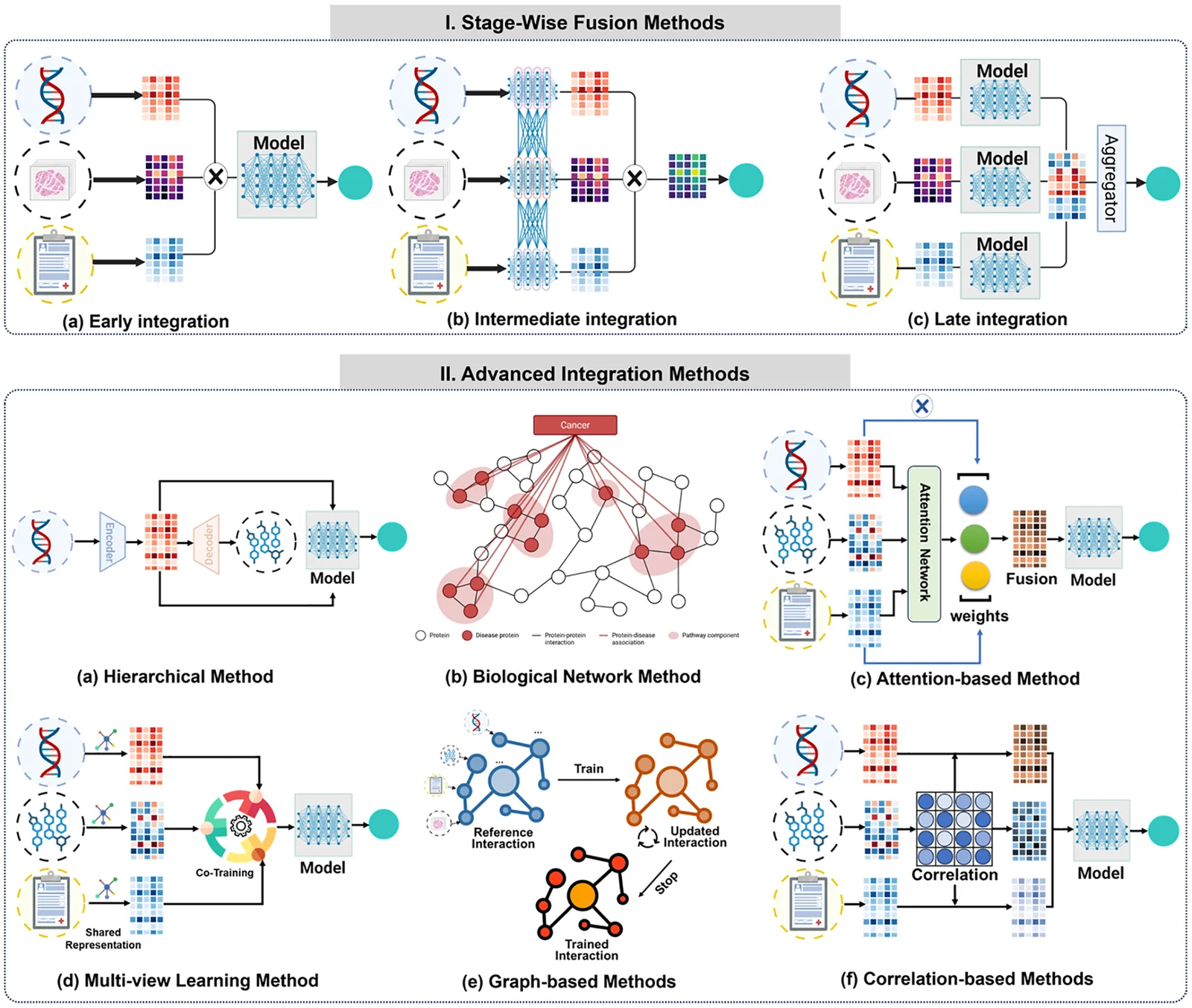
Methods of multi-modal data integration: **a** Stage-wise fusion; **b** advanced integration methods

**Fig. 5:**
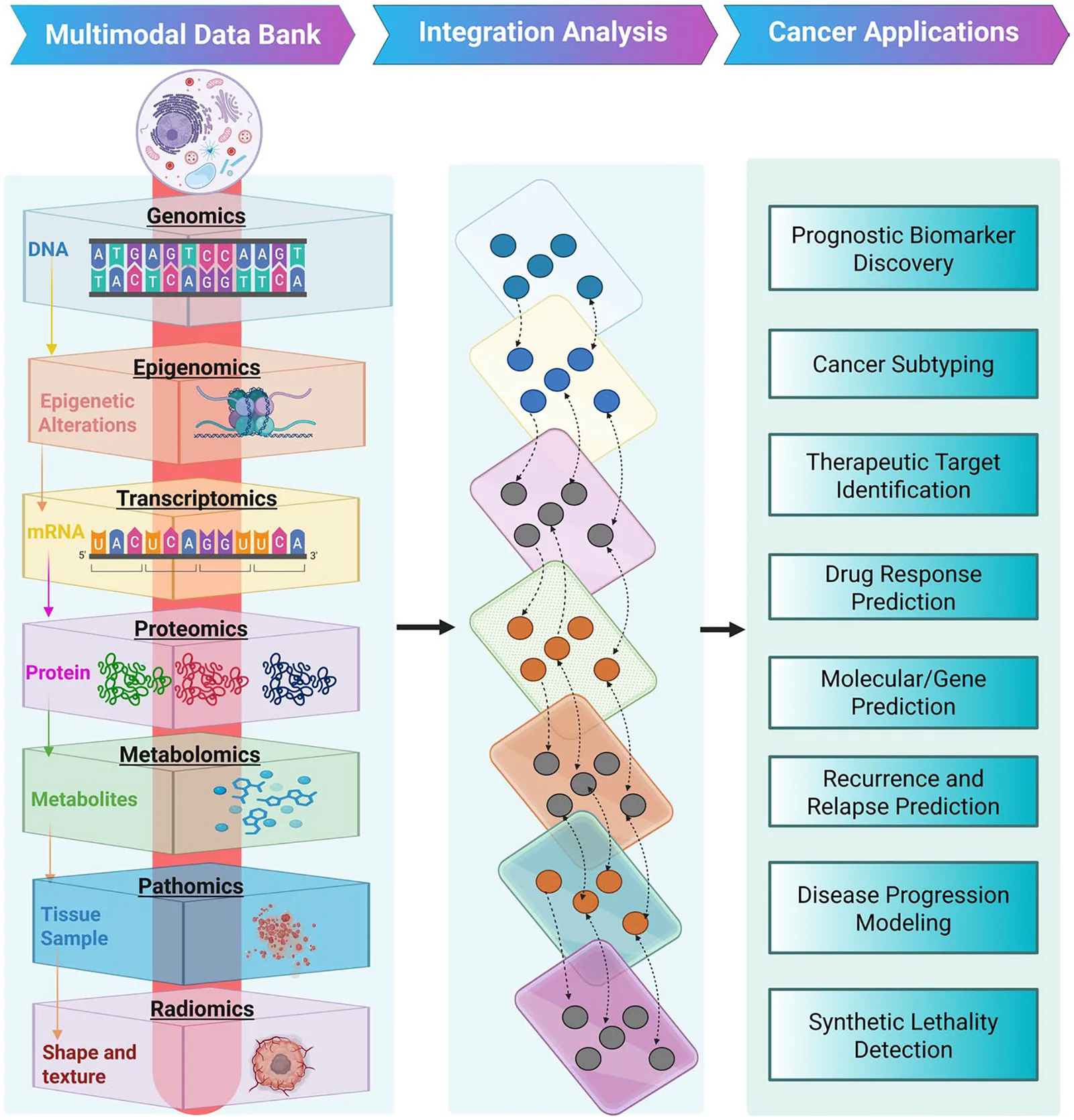
Multi-modal integration pipeline from modality data bank to cancer applications

**Fig. 6:**
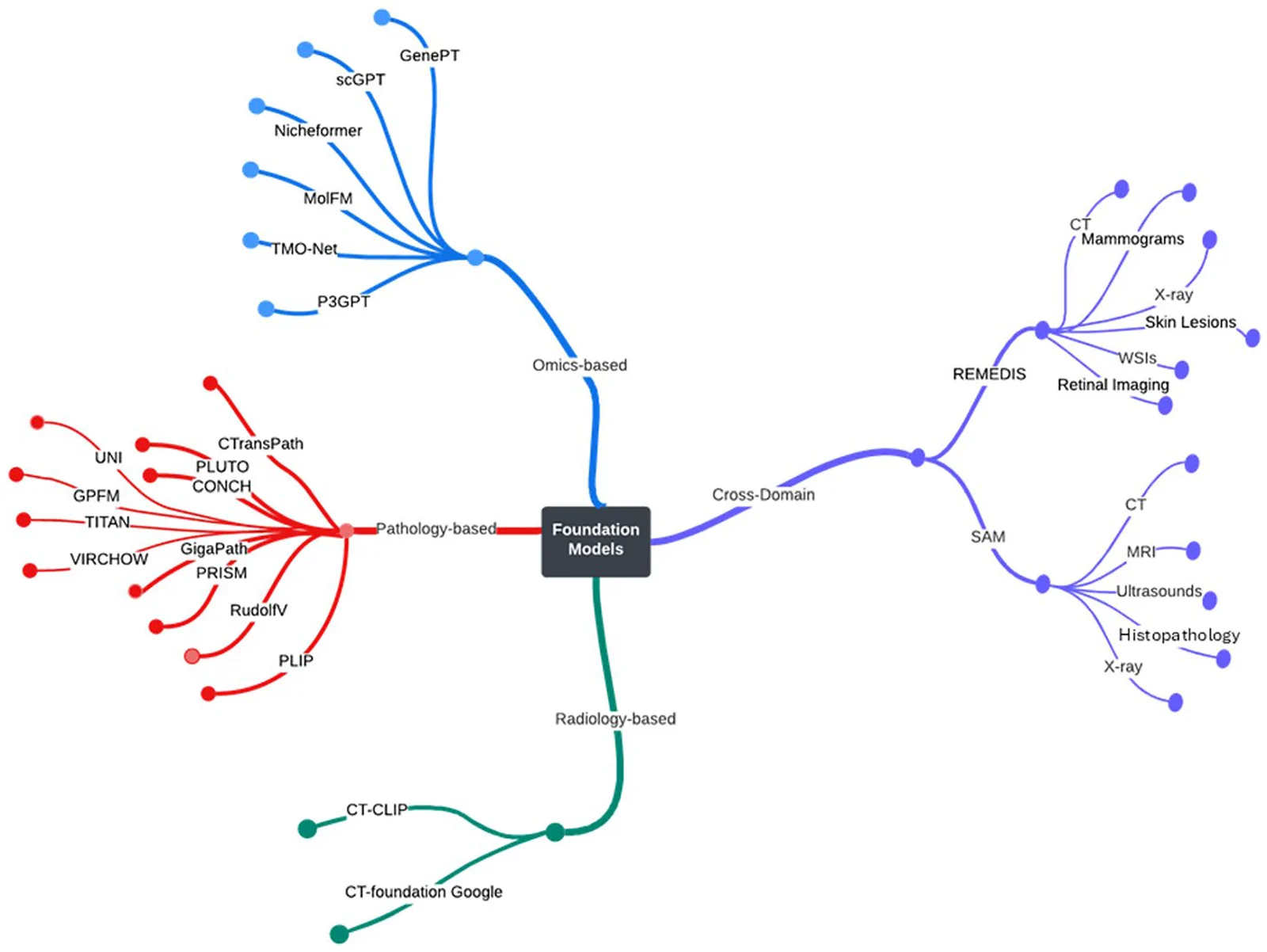
A proposed taxonomy for foundation models in multimodal cancer research. The framework delineates four key domains: (i) unimodal models for omics, (ii) pathology, and (iii) radiology, and (iv) integrative models designed for cross-domain data

**Fig. 7:**
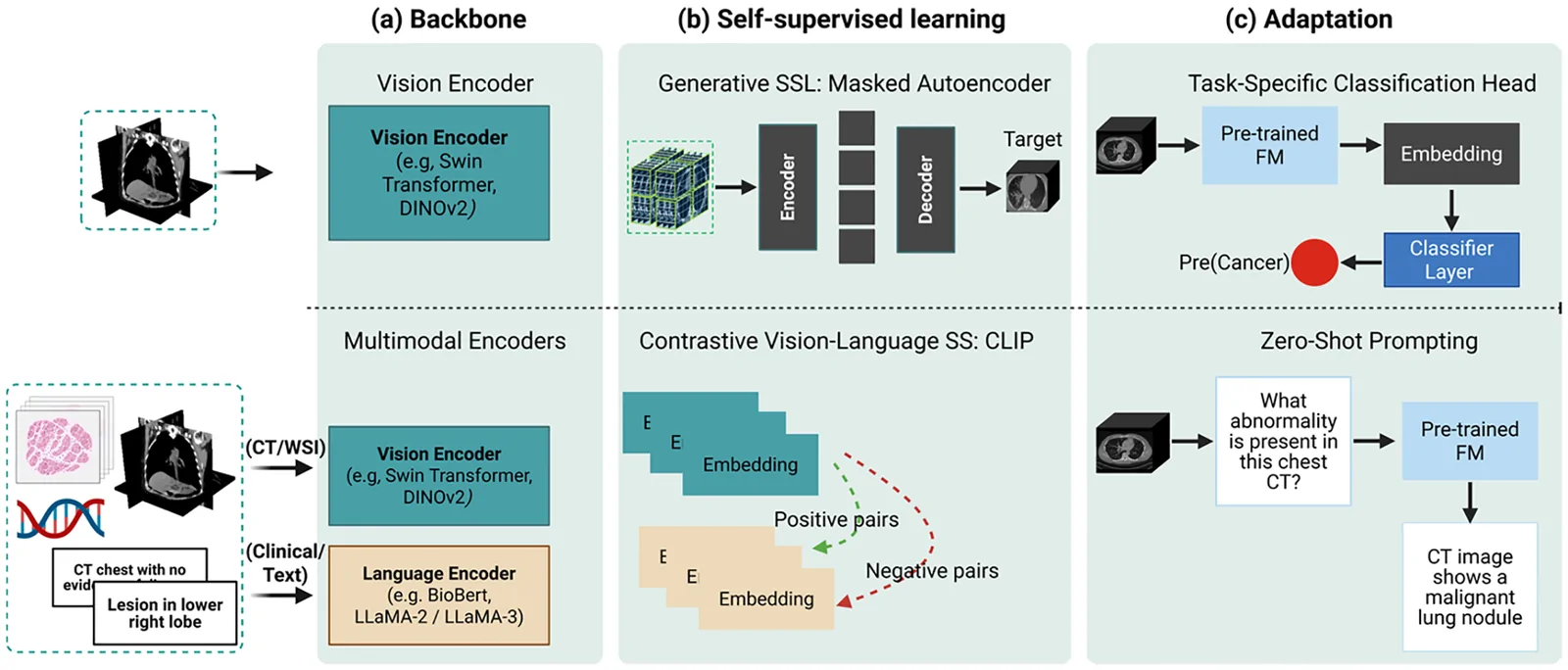
Algorithmic design of Cancer multimodal FMs. **a** Domain-specific backbones, including unimodal vision encoders and multimodal vision–language encoders, **b** Self-supervised pretraining using generative masked-autoencoder objectives and contrastive vision–language learning, and (**c**) Downstream adaptation via task-specific heads or zero-shot prompting

**Fig. 8:**
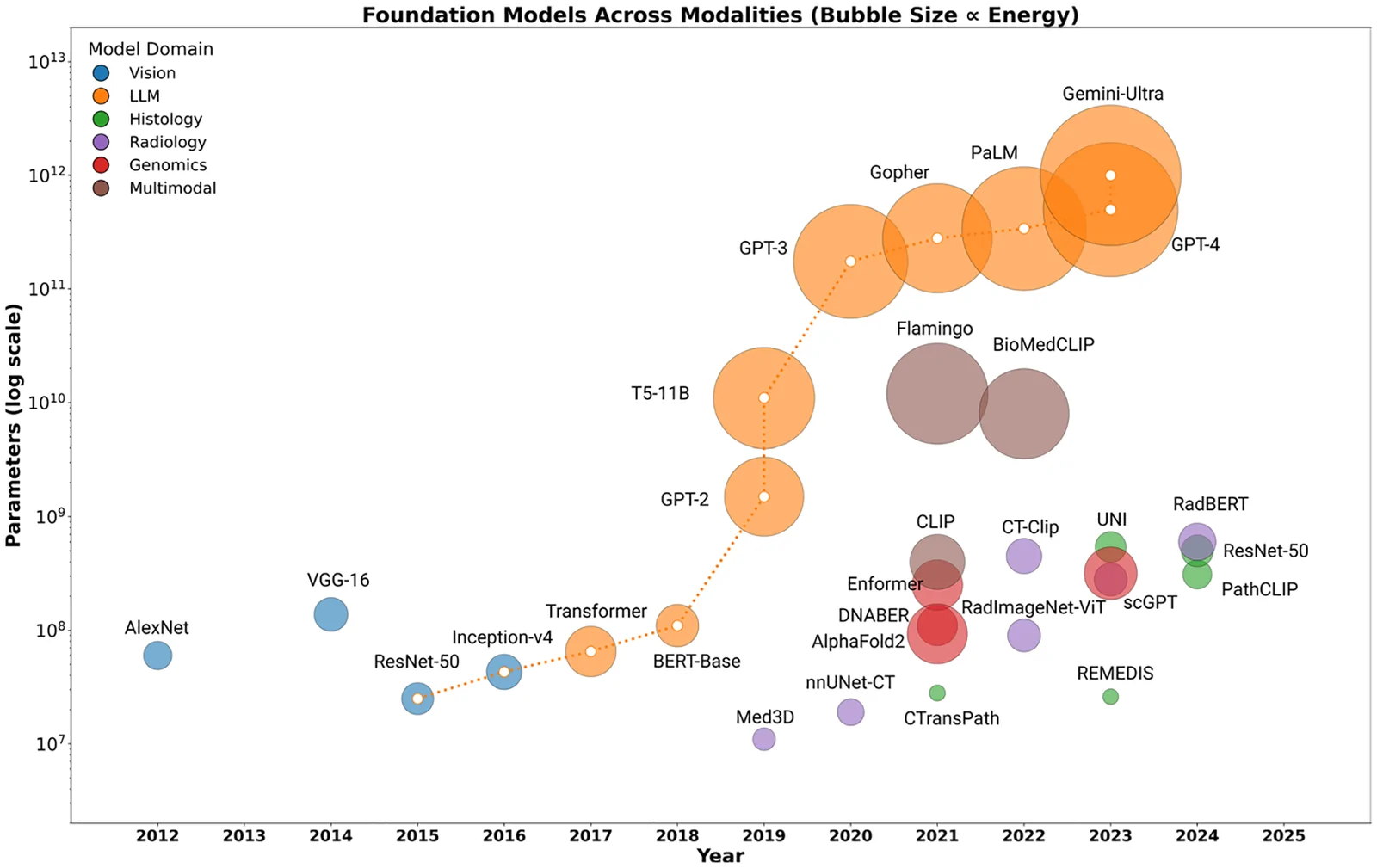
Growth of foundation models across modalities (bubble size ∝ relative compute/energy proxy). Parameter values and release years are taken from original model papers and technical reports. Bubble area is proportional to an order-of-magnitude proxy for per-inference energy cost, computed from reported or commonly cited FLOP estimates under a fixed hardware efficiency assumption. The dotted line summarizes the approximate exponential increase in parameter count over time; bubble sizes are intended as qualitative, not exact, energy comparisons

**Fig. 9:**
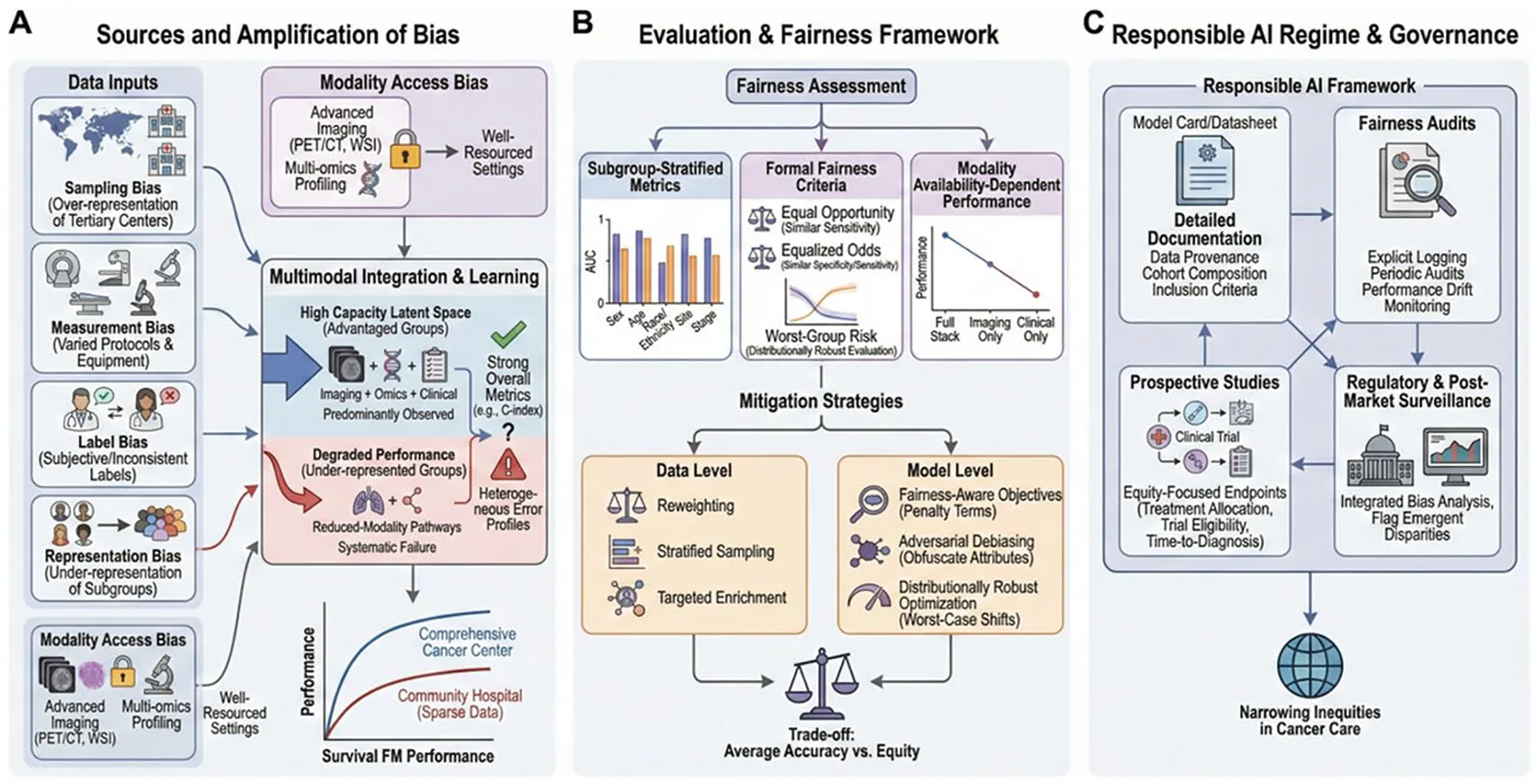
Algorithmic Bias, Fairness, and Health Equity in Multimodal Oncology FMs

**Fig. 10:**
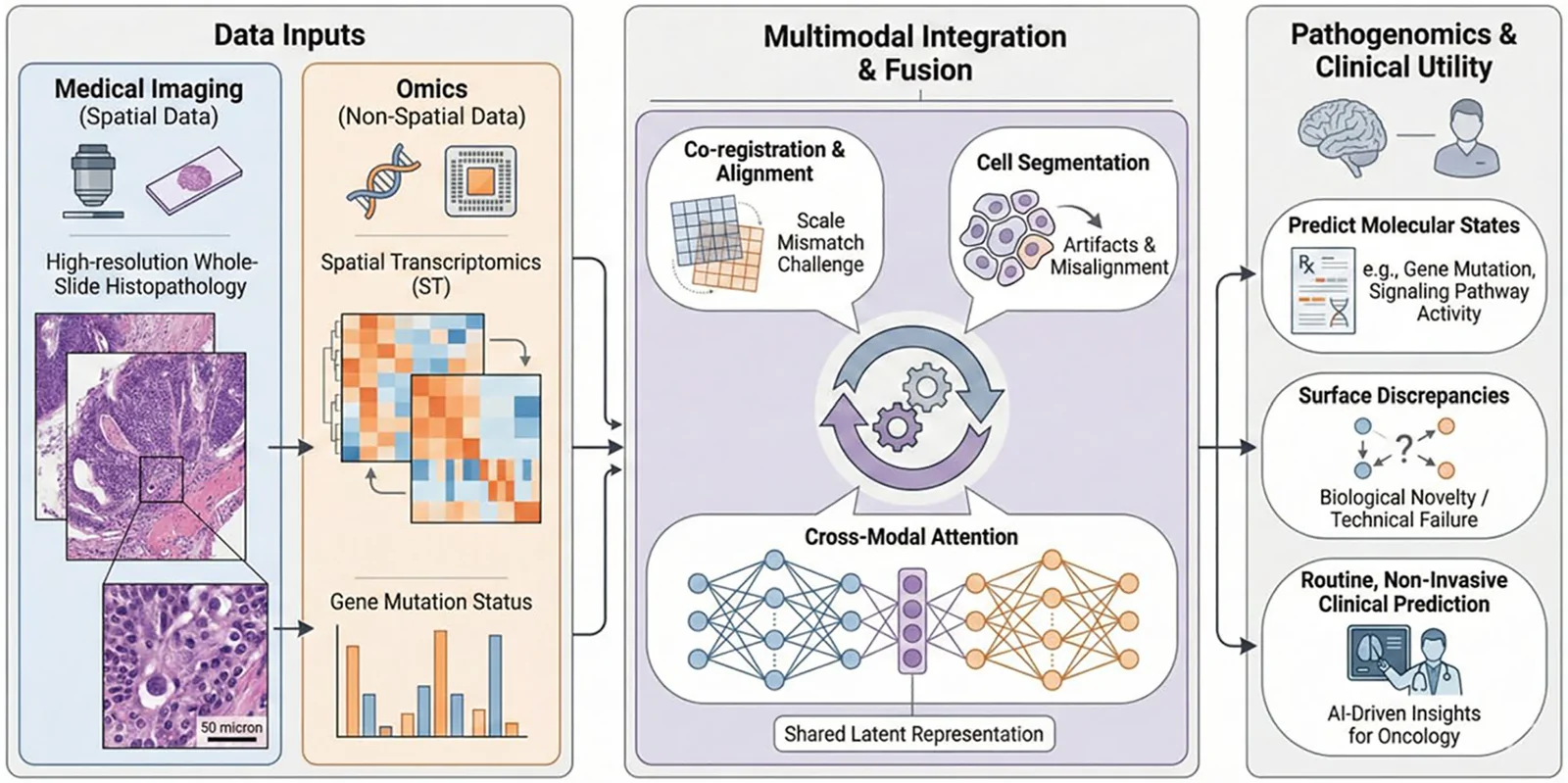
Schematic overview of a multimodal pathogenomics pipeline

**Table 1:** Comparative strengths, limitations, and recommended use-cases for multimodal integration strategies

Integration Method	Strengths	Limitations	Most Appropriate Use-Cases
Early Fusion (Feature-Level)	Simple; captures cross-modal interactions; minimal architectural complexity	High dimensionality; prone to overfitting; requires complete data; low interpretability	Large, aligned datasets; when cross-modal feature interactions are important
Intermediate Fusion (Representation-Level)	Reduces dimensionality; modality-specific representation learning; scalable	Loss of interpretability; dependent on learned representations; complex tuning	High-dimensional heterogeneous data requiring joint latent representations
Late Fusion (Decision-Level)	Robust to missing modalities; modular; more interpretable	Cannot model feature-level synergies; risk of modality dominance	Clinical settings with heterogeneous or incomplete multimodal data
Hierarchical Methods	Leverage biological priors; interpretable regulatory flow; mechanistically grounded	Dependent on accuracy of biological pathways; limited flexibility; error propagation	When regulatory directionality (DNA→RNA→protein) is known and meaningful
Graph-Based Integration	Captures network topology; pathway-aware; powerful relational modeling	Dependent on curated network quality; computationally intensive; complex interpretation	Pathway analysis, network biology, regulatory module discovery
Multi-View Learning	Uses complementary perspectives; encourages consensus; noise-robust	Fails with modality disagreement; may suppress unique signals; require sample matching	Complementary omics layers describing shared biology
Correlation-Based Methods (CCA, PLSR)	Interpretable; identifies shared variation; statistically grounded	Primarily linear; sensitive to noise; requires paired data	Linking omics layers; shared covariance discovery; exploratory analysis
Imaging–Omics Fusion (Spatial & Non-Spatial)	Captures spatial context; phenotype localization; attention improves interpretability	Requires large imaging datasets; alignment issues; computationally heavy	Radiopathomics, pathomics, spatial biomarker discovery
Multimodal FMs	Highly flexible; handle unpaired/incomplete data; powerful joint representations	High computational cost; reduced interpretability; requires large pretraining corpora	Large-scale multimodal diagnosis, prognosis, and biomarker discovery

**Table 2:** Comprehensive Multi-Modal data repositories

DATA REPOSITORY	WEB LINK	DISEASE	Types Of Multi-Omics Data Available
The Cancer Genome Atlas (TCGA)	https://www.cancer.gov/tcga	Multiple cancer types (e.g., lung, breast, prostate, colorectal, etc.)	Genomics, Transcriptomics, Epigenomics, Proteomics, Clinical metadata.
Gene Expression Omnibus (GEO)	https://www.ncbi.nlm.nih.gov/geo/	Various cancer types (breast, lung, colorectal, leukemia, prostate, melanoma, ovarian, etc.)	Genomics, Transcriptomics
Metabolomics Workbench	https://www.metabolomicsworkbench.org/	Breast, lung, colorectal, prostate, hematologic malignancies, ovarian and other cancer types	Metabolomics (LC-MS, GC-MS, NMR)
Human Protein Atlas (HPA)	https://www.proteinatlas.org/	Over 20 major cancers (breast, lung, colorectal, prostate, liver, ovarian, glioma, etc.)	Proteomics, transcriptomics (RNA-seq quantification), spatial proteomics, single-cell RNA-seq, clinical survival data
EGA (European Genome-Phenome Archive)	https://ega-archive.org/	Breast, colorectal, prostate, lung, hematologic malignancies and other cancers.	Genomics, Epigenomics, Clinical metadata
ArrayExpress	https://www.ebi.ac.uk/arrayexpress/	Breast, lung, colorectal, prostate, hematologic malignancies, ovarian, melanoma and other cancers	Transcriptomics, Epigenomics
cBioPortal	https://www.cbioportal.org/	Multiple cancer types (e.g., breast, prostate, glioblastoma, leukemia)	Genomics, Transcriptomics, Clinical metadata.
ProteomicsDB	https://www.proteomicsdb.org/	Various (e.g., cancer-focused proteomic and PTM profiles, drug-target interaction, disease biomarker and therapeutic target discovery)	Proteomics
ENCODE (Encyclopedia of DNA Elements)	https://www.encodeproject.org/	Breast, lung, colorectal, hematologic and other cancer cell lines and tissues	Genomics, Epigenomics
Synapse (Sage Bionetworks)	https://www.synapse.org/	Diverse cancers (breast, lung, colorectal, prostate, ovarian, hematologic malignancies, glioma)	Genomics, transcriptomics, proteomics, epigenomics, clinical metadata
MetaboLights	https://www.ebi.ac.uk/metabolights/	Breast, lung, gastric, renal, liver, colorectal, prostate, and multiple myeloma	Metabolomics and associated metadata
LINCS (Library of Integrated Network-based Cellular Signatures)	https://lincsproject.org/	Cancer cell lines (leukemia, lymphoma, breast, lung, colon, prostate, and brain cancer)	Transcriptomics, Proteomics, Metabolomics
GDC (Genomic Data Commons)	https://gdc.cancer.gov/	Multiple cancer types (e.g., colorectal, lung, breast)	Genomics, Transcriptomics, Clinical metadata
HuBMAP (Human Biomolecular Atlas Program)	https://hubmapconsortium.org/	Reference baseline for human tissues (healthy organs; enables cancer atlas comparisons)	Transcriptomics, Proteomics, Spatial Data
Cancer Proteome Atlas (TCPA)	https://tcpaportal.org/	Multiple cancer types (e.g., lung, colorectal, ovarian)	Proteomics, Clinical metadata
ImmPort	https://www.immport.org/	Cancer immunotherapy and tumor microenvironment studies (melanoma, lung, breast, hematologic malignancies)	Genomics, Transcriptomics, Immunomics
PRIDE Archive	https://www.ebi.ac.uk/pride/	Pan-cancer: breast, lung, colorectal, prostate, glioma, leukemia, ovarian, and more.	Proteomics
Open Targets Platform	https://www.opentargets.org/	Multiple cancers (breast, lung, colorectal, prostate, melanoma, leukemia, ovarian, etc.)	Genomics, Clinical
BioGRID (Biological General Repository for Interaction Datasets)	https://thebiogrid.org/	Various (e.g., breast, lung, colorectal, prostate, hematologic, ovarian, etc.)	Protein-protein interactions; genetic interactions (e.g., synthetic lethality)
ICGC ARGO (Accelerating Research in Genomic Oncology)	https://www.icgc-argo.org/	Multiple cancer types (e.g., bladder, breast, colorectal, gastric, head and neck, lung, ovarian)	Genomics, Transcriptomics
TCIA (The Cancer Imaging Archive)	https://www.cancerimagingarchive.net/	Multiple cancer types (e.g., lung, brain, liver)	Pathomics, Radiomics, Genomics, Clinical metadata
CPTAC (Clinical Proteomic Tumor Analysis Consortium)	https://hupo.org/Clinical-Proteome-Tumor-Analysis-Consortium-(CPTAC)	Multiple cancer types (e.g., breast, kidney, lung)	Pathomics, Genomics, Proteomics, Clinical metadata
Pan-Cancer Atlas	https://gdc.cancer.gov/about-data/publications/pancanatlas	Multiple cancer types (e.g., pancreatic, breast, lung)	Genomics, Transcriptomics, Proteomics
BioSamples	https://www.ebi.ac.uk/biosamples/	Breast, lung, colorectal, prostate, ovarian, melanoma, hematologic malignancies.	Metadata, genomics, transcriptomics, proteomics, metabolomics and epigenomics.
Recount2	https://jhubiostatistics.shinyapps.io/recount/	Diverse TCGA cancer cohorts (breast, lung, colorectal, prostate, glioma, etc.)	Transcriptomics
Xena UCSC	https://xenabrowser.net/	Multiple cancer types (e.g., lung, breast, pancreatic)	Genomics, Transcriptomics, Epigenomics, Clinical metadata
Zenodo	https://zenodo.org/	Various cancer types (depends on collection; includes multi-omics and imaging cohorts)	Multi-omics (genomics, transcriptomics, proteomics, metabolomics), radiomics/pathomics, and clinical metadata (dataset-specific).
Human Tumor Atlas Network (HTAN)	https://humantumoratlas.org/	Multiple solid tumors and hematologic malignancies (multi-organ tumor atlases)	Single-cell and spatial transcriptomics, proteomics, pathology and advanced imaging, clinical metadata
Hest-1 K	https://github.com/mahmoodlab/HEST	Cancer cohorts with matched H&E WSIs and spatial omics (tumor-type specific)	Pathomics (WSIs), spatial transcriptomics/proteomics, and associated clinical metadata

**Table 3:** Conceptual overview of multimodal integration strategies, representative methodological families, and their typical objectives, interpretability characteristics, and input requirement

Integration Strategy	Representative Method Types	Typical Objectives	Interpretability	InputRequirements
Early Fusion	MOFA/MOFA+, iCluster, matrix, tensor, factorization	Unsupervised subtyping, pathway-level biomarker discovery	High (explicit latent factors)	Requires matched modalities with minimal missingness
Intermediate Fusion	Attention-based fusion, VAEs, CCA, DCCA, DGCCA	Prognosis, risk stratification, therapy-response prediction	Moderate (latent spaces + attention weights)	Supports heterogeneous modalities; tolerant to partial missingness
Late Fusion	Ensemble learning across modality-specific models	Diagnostic classification, workflow augmentation, clinical decision support	Low to moderate (model-level interpretation only)	Robust to missing modalities; flexible combinations
Graph-Based Methods	GNNs using PPI, GRN, or biologic interaction networks	Therapeutic vulnerability prediction, mechanism-informed modeling	Moderate to high (node or edge importance, topology)	Requires curated biological networks or inferred graphs
Spatial or Non-Spatial Imaging–Omics Fusion	Radio-pathomic, pathomic attention-based fusion	Prognosis, biomarker estimation, spatial phenotype discovery	Moderate (spatial attention maps)	Imaging plus optional clinical or molecular data
Multimodal Foundation Models	Unified transformer architectures for omics + imaging +text	Diagnosis, prognosis, biomarker discovery	Often complex (attention or saliency improving)	Handles heterogeneous, incomplete, or partially paired inputs

**Table 4:** Meta-analysis and data extraction of the selected studies

Ref	Year	Methods	Data Types	Integration Type	Validation	Main Results	Aim	Source Code
(Wang et al. 2024a)	2024	TMO-Net: Variational autoencoders with cross-fusion for joint embeddings	CNV, mRNA, mutation, methylation	Molecular multiomics (genomics, transcriptomics, methylation)	5-fold CV, CPTAC external	F1:0.75–0.92; C-index:0.60–0.8	Joint embedding for prognosis	https://github.com/FengAoWang/TMO-Net
(Zhang et al. 2024a)	2024	Radiomics & deep features (ResNet50), LASSO & ML classifiers	WSI, MRI (Pathomics, Radiomics)	Image-based multimodal	Train/val split, 5-fold CV	AUC composite model: 0.85–0.93	Early diagnosis prostate bone metastasis	N/A
(Ross et al. 2024)	2024	Multimodal AI with WSIs & clinical data	WSIs, clinical (PSA, Gleason)	Multimodal clinical/image	Clinical trial validation	HR for DM: 2.33; PCSM: 3.54	Predict clinical outcomes (PCa)	N/A
(Hu et al. 2024)	2024	ML for biochemical recurrence prediction	MRI, WSIs, clinical (PSA, TNM)	Multimodal clinical/image	363 patients (train/test)	C-index:0.860; AUC:0.911 (3-year BCR)	Predict BCR post-prostatectomy	N/A
(Tan et al. 2024b)	2024	Pathomics (ResNet-18), Logistic Regression	Pathological slides, clinical markers	Pathological & clinical	Internal dataset (*n* = 267)	AUC combined:0.907 train,0.814 test	Colorectal cancer staging	N/A
(Sun et al. 2024)	2024	AI-LLMs integrated with CNN/Transformer	Clinical data, CT images	Text & image fusion	163 patients, 64 tests	Extraction accuracy:87–97%; AUC:0.89 max	Survival prediction bladder cancer	N/A
(Zemke et al. 2023)	2023	ML for cis-regulatory elements	Multi-omics (single cell)	Multi-omics molecular	Cross-species validation	Accuracy:0.65–0.90; correlation:0.8–0.88	Predict epigenetic conservation	https://github.com/ejarmand/comparative_epigenomic_motor_cortex
(Zhu et al. 2023a)	2023	Geometric Graph Neural Networks (GGNN)	RNA-Seq, CNA, methylation	Multi-omics molecular	Multiple cancers (TCGA)	High C-index (except colon cancer)	Survival prediction	(https://github.com/MSK-MOI/GGNN
(Qi et al. 2023)	2023	CNNs for PET/CT quality assessment	PET/CT imaging	Imaging	Internal dataset (173 patients)	Kappa:0.78–0.80; ICC:>0.75	Automated PET/CT quality assessment	N/A
(Chang et al. 2023)	2023	Radiomics via SDCT	Imaging (SDCT sequences)	Imaging multimodal	Internal dataset (176 patients)	AUC combined:0.961 train,0.944 test	Predict lung adenocarcinoma invasiveness	N/A
(Huang et al. 2023)	2023	Contrastive Learning (PLIP)	Pathology images/text	Visual-language multimodal	Zero-shot evaluation, fine-tuned	F1:0.856–0.927; Recall@10:0.557	Visual-language foundation pathology	https://github.com/PathologyFoundation/plip
(Zhu et al. 2023b)	2023	Multimodal spatial attention (SAMMS)	Multi-omics & histopathological	Spatial attention multimodal	5-fold CV (TCGA)	C-index:0.843; AUC:0.782 (LGG)	Cancer survival prediction	N/A
(Pu et al. 2022)	2022	GNNs for therapeutic effects prediction	Protein interactions, gene expression	Molecular multi-omics	CCLE, LINCS	AUC: ~0.8	Predict kinase inhibitors effect	https://github.com/pulimeng/CancerOmicsNet
(Tsai et al. 2023)	2022	Vision Transformer & multi-instance learning	WSIs, multi-omics	Image & molecular multimodal	Independent cohorts & TCGA	AUC:>0.85; F1:>0.80	Prognosis colorectal cancer	https://github.com/pulimeng/CancerOmicsNet.
(Yu et al. 2021)	2021	MRI radiomics & SVM classifiers	MRI, clinical-pathologic	Imaging & clinical	Multicenter dataset	AUC:0.90–0.93	ALN metastasis prediction	https://github.com/ZifanHe/Ebiomedicine.g.it
(Feng et al. 2022)	2022	Radiopathomics (RAPIDS)	MRI, WSIs	Image multimodal	Multicenter observational	AUC:0.81–0.86	Predict chemotherapy response	Radiopathomics (RAPIDS)
(Peng et al. 2021)	2021	CNN (VGG16, ResNet50)	Colposcope images	Image multimodal	Internal dataset	Accuracy:86.3%	Cervical lesion classification	N/A
(Fu et al. 2021)	2021	U-Net with multimodal attention	PET/CT images	Imaging multimodal	5-fold CV	Dice:71.44–62.26	Tumor segmentation	N/A
(Ding et al. 2020)	2021	CNN & Word2Vec fusion	Endoscopy images & text	Multimodal fusion	Dataset split	Accuracy:95.3%; sensitivity:90.2%	UGI cancer screening	https://github.com/netflymachine/SCNET
(Yan et al. 2021)	2021	Richer Fusion Network	Pathology images & EMR	Multimodal fusion	Dataset split	Accuracy:92.9%	Breast cancer classification	N/A
(Guo et al. 2020)	2020	MSReg with 3D ResNeXt	MRI & Ultrasound	Image registration	NIH clinical trial (*n* = 679)	Error reduced up to 62%	Prostate cancer biopsy guidance	N/A
(Cao et al. 2020)	2020	EPLA with MIL & ResNet-18	Histopathology, multi-omics	Multimodal fusion	TCGA, Asian-CRC	AUC:0.8848 – 0.8504	Predict MSI colorectal cancer	https://github.com/yfzon/EPLA
(Ding et al. 2023)	2023	Transformer-based PathOmics	WSI, mRNA, CNV, DNA methylation	Multimodal embeddings	TCGA COAD, TCGA-READ	AUC:0.56–0.75	Survival prediction in colon cancer	https://github.com/Cassie07/PathOmics
(Peneder et al. 2021)	2021	ML classifiers for cfDNA analysis	cfDNA liquid biopsy data	Integrativeanalysis	10-fold CV	AUC:0.76–0.97	Pediatric sarcoma diagnosis	https://medical-epigenomics.org/papers/peneder2020_f17c4e3befc643ffbb31e69f43630748/
(Mu et al. 2021)	2021	DL (SResCNN) for PD-L1 prediction	PET/CT, clinical data	Multimodal fusion	Internal, external cohorts	AUC:0.82–0.89; accuracy:77.7%–81.7%	PD-L1 prediction NSCLC	N/A
(Song 2021)	2021	XGBoost-based radiomics	PET/CT imaging	Imaging multimodal	Internal dataset	Sensitivity:90.9%; specificity:71.4%; accuracy:80%	Predict ALN metastasis (IDC)	N/A
(Poirion et al. 2021)	2021	Ensemble DL & ML (DeepProg)	Gene expression, mutation, methylation	Multi-omics integration	TCGA, GEO datasets	C-index:0.68–0.80	Prognosis prediction	https://github.com/lanagarmire/DeepProg
(Papp et al. 2021)	2021	Ensemble random forest radiomics	PET/MRI imaging (Ga-PSMA-11, ADC, T2w)	Imaging multimodal	Internal dataset (52 patients)	AUC:0.86–0.94; accuracy:81%–91%	Risk assessment prostate cancer	N/A
(Peneder et al. 2021)	2021	LIQUORICE ML for cfDNA patterns.	Genomic, epigenomic (cfDNA)	Integrative analysis	ENCODE, TCGA	AUC:0.97, Sens:85% @ 100% Spec	cfDNA-based pediatric cancer detection (EwS/sarcoma)	https://liquorice.readthedocs.io/en/latest/intro.html?utm_source=chatgpt.com
(Liu et al. 2020)	2020	IOUC-3DFCNN with Haar-like fusion	MRI (T1, T1GD, T2, Flair)	Multimodal auto-context	BRATS 2017 & 2013 datasets	DICE:0.70–0.89; Recall:0.79–0.91	Brain tumor segmentation (Gliomas)	N/A
(Boehm et al. 2022)	2022	CPH with L1/L2 regularization	WSI, CT, clinicogenomics	Late multimodal fusion	Train-test split (444 patients)	Significant correlation with GVHD, OS:81%, PFS:76%	Predict GVHD after bone marrow transplant	https://github.com/kmboehm/onco-fusion
(McCurdy et al. 2022)	2022	CART analysis on immunophenotypic, proteomic, clinical data	Immunophenotypic, proteomic, clinical	Multimodal integration	Two trials (145 patients)	Significant correlation with GVHD, OS:81%, PFS:76%	Predict GVHD after bone marrow transplant	N/A
(Chen et al. 2020)	2020	Pathomic Fusion with gating-attention	Histology, genomics (CNV, RNA-seq)	Multimodal fusion	15-fold CV	Accuracy:85–95%, ROC-AUC:0.92–0.98, C-Index:0.72–0.85.	Survival outcome prediction	https://github.com/mahmoodlab/PathomicFusion
(Motzer et al. 2020)	2020	NMF clustering, DGE, pathway & co-occurrence analysis	Transcriptomic, somatic genomic	Multi-omics clustering	Internal validation, TCGA	Identified prognostic mutations and therapy sensitivity	Subtyping RCC and therapy response	N/A

**Table 5:** Summary of cancer research studies employing Foundation models in oncology

Omics-based Foundation Models						
Foundation Model	Year	Objective/Purpose	Training Modality	Training Cohorts	Training Method	Downstream Tasks
GET (Fu et al. 2025)	2025	Models regulatory grammar across human cell types	Chromatin accessibility + DNA sequence	213 human fetal and adult cell types	Transformer-based FM using chromatin accessibility data and sequence information	Gene expression, TF interactions, regulatory element and response prediction
OmniCLIP (Chen et al. 2025)	2025	Visual omics FM linking WSIs with spatial transcripts via contrastive learning	WSIs + STs	2.2 million paired tissue images from 1,007 samples across 32 organs using 10× Visium spatial data	CLIP-based FM using contrastive learning, by using transcriptomic data into sentences with a dual encoder architecture	Tissue alignment, annotation, decomposition, retrieval, spatial gene prediction.
scGPT (Cui et al. 2024)	2024	Distills critical biological insights concerning genes and cells	Multi-Omics	Single-Cell Sequencing Data over 33 million cells	A generative pretrained transformer	Cell type annotation, multi-omics integration, response prediction, gene network inference
Nicheformer (Schaar et al. 2024)	2024	Cell embeddings reflect local microenvironment context.	Single-Cell STs + Spatially Resolved Transcriptomics	110 M scRNA-seq + spatial cells (MER-FISH, Xenium, CosMx, ISS)	Transformer-based models use gene-rank tokenization with a 12-layer, 16-head transformer.	Spatial density prediction, spatial label prediction, cross-modal transfer
MolFM (Luo et al. 2023)	2023	Multimodal FM learning from molecular structures, texts, and knowledge graphs.	Molecular structures+knowledge graphs + and biomedical texts	5k structures, 37 M text paragraphs, 49k-entity KG (3.2 M relations)	Cross-modal attention aligning molecular modalities while maintaining global knowledge.	Cross-modal retrieval, molecule captioning, text-based molecule generation, and molecular property prediction
GenePT (Chen and Zou 2023)	2023	FM representing genes and cells using LLM-derived embeddings	NCBI gene database.	Use GPT-3.5 to create embeddings from the NCBI text descriptions of genes.	LLM ChatGPT (GPT-3.5)	Gene functionality classification, gene property prediction, GGI and PPI prediction, Cell Type Annotation
TMO-Net (Wang et al. 2024c)	2024	Multi-omics integration for pan-cancer with missing-modality support.	Gene Mutation, mRNA Expression, CNV, DNA Methylation Profiles data	TCGA which includes 8,174 samples	multi-task pretraining with cross-fusion module and self-supervised contrastive learning	cancer subtyping, metastasis prediction, drug response prediction, prognosis prediction
Precious3-GPT (P3GPT) (Galkin et al. 2024)	2024	Multimodal transformer FM for aging and drug discovery using multi-species multi-omics data.	RNA-seq (GTEx, ARCHS4, LINCS) + DNA methylation and proteomics datasets.	1.2 M samples across 63k+ biological entities (multi-species)	Prompt-driven multimodal transformers (omics + text)	Age prediction, Target identification, drug sensitivity prediction, gene annotation
DNA-BERT-2 (Zhou et al. 2023)	2023	Multi-species genomics FM overcoming k-mer tokenization limits	DNA Sequences	Multi-species genome dataset combining 36 datasets across 9 tasks (70–10,000 bp)	Uses Byte Pair Encoding (BPE) + transformer for long multi-species genomes	Genomic variant prediction, cancer mutation assessment, genomics analysis, cancer genomics applications
Pathology-based Foundation Models						
Foundation Model	Year	Objective/Purpose	Training Modality	Training Cohorts	Training Method	Downstream Tasks
MUSK (Xiang et al. 2025)	2025	Multimodal transformers for predicting cancer by integrating pathology images with clinical text data through cross modal learning	WSIs + Text	50 million pathology images paired with 1 billion pathology related text descriptions from multiple cancer centers and clinical databases.	Leverages unified mask modeling approach to handle unpaired multimodal data with cross modal attention, contrastive learning between image and text modality	Disease level survival prediction, cancer subtyping, biomarker identification, treatment response prediction
Prov-Giga-Path (Xu et al. 2024a)	2024	Clinical grade foundation model for digital pathology for cancer diagnosis and prognosis	WSIs	1.3 billion 256 × 256 pathology image tiles in 171,189 whole slides 30,000 patients covering 31 major tissue types	Tile level training pretraining using DINOv2 (Oquab et al. 2023), slide-level training using masked level autoencoder, vision language pretraining with clinical reports	Biomarker prediction, mutation status prediction, survival analysis, cancer subtyping
PathCHAT (Lu et al. 2024b)	2024	Vision language AI assistant for human pathology - multimodal generative AI copilot	WSIs + Text	456,000 different visual language instructions consisting of 999,202 question and answers	Pretrained large language model and fine-tuned on visual language instructions	Pathological query assistance, diagnostic support, morphological analysis, differential diagnosis
BEPH (Yang et al. 2025a)	2025	Uses self-supervised learning (SSL) for generalizable cancer diagnosis and survival prediction	WSIs	11 million histopathological images from multiple cancer subtypes	SSL for learning meaningful representations from histopathological images	Patch-level cancer prediction, cancer classification, survival prediction for cancer types
CHIEF (Wang et al. 2024b)	2024	Uses weakly-supervised machine learning to extract pathology imaging features	WSIs	60,530 WSIs consisting of 19 anatomical sites	Unsupervised pretraining for tile level feature identification and weakly supervised pretraining for whole slide pattern recognition	Cancer cell detection, prognostic prediction, tumor identification, genomic prediction
PLUTO (Juyal et al. 2024)	2024	The generation of task-agnostic embeddings.	WSIs	Pretrained on a dataset of 195 million tiles from 158, 852 WSIs.	DINO (Caron et al. 2021) + iBOT SSL with MAE and Fourier losses to preserve high-frequency detail	Instance segmentation, tile classification, slide-level class, and survival analysis.
UNI (Chen et al. 2024a)	2024	Interpreting pathology images; surgical pathology	WSIs	100 million images from over 100,000 diagnostic WSIs across 20 major sub-types	SSL and supervised fine-tuning	Slide classification, ROI classification and retrieval, cell type segmentation, and slide prototyping.
GPFM (Ma et al. 2024)	2024	Improve the generalization capability of pathology FMs across a wide range of clinical tasks.	WSIs	86,000 WSIs across 34 major tissue types	Expert and self-knowledge Distillation	Slide Classification, ROI analysis, Image retrieval, Pathological Images VQA, and Report generation.
TITAN (Ding et al. 2024)	2024	Incorporates multimodal learning by aligning visual features of pathology slides with synthetic captions and clinical reports.	WSIs + Text + synthetic Region-of-Interest (ROI) Captions	335,645 WSIs with 423,122 synthetic captions at the region-of-interest, and 82,862 clinical pathology reports.	Three-stage pretraining: SSL on WSIs, then alignment with captions and clinical reports.	Survival Prediction, Morphological Classification, Molecular Classification, Grading Tasks, Cross-Modal Retrieval, Pathology Report Generation
VIRCHOW (Vorontsov et al. 2024)	2024	To develop clinical-grade FM for digital pathology by enabling the detection of both common and rare cancers	WSI	1.5 million H&E-stained WSIs from around 100,000 patients	The model was trained using the DINO v2 framework (Oquab et al. 2023), a self-supervised multi-view student-teacher approach	Pan-Cancer Detection and subtyping, tile-level prediction, survival analysis,
GigaPath (Xu, 2024b)	2024	GigaPath is to develop to generalize effectively across diverse real-world clinical settings	WSIs	1.3 billion 256 × 256 image tiles from 171,189 whole slides.	Three-stage training, which includes tile-level pretraining: Using DINOv2 (Oquab et al. 2023), slide-level training using a masked autoencoder and LongNet, and VLM pretraining.	17 pathomics tasks, Mutation Prediction, and zero-shot cancer subtyping
RudolfV (Dippel et al. 2024)	2024	To develop a pathology foundation model of diverse pathologist-guided curation.	WSIs	134,000 slides from 34,103 cases grouped by semantic similarity, and clustered tiles to ensure meaningful pattern learning.	Trained using DINOv2 (Oquab et al. 2023) for SSL	Slide classification, reference search, IHC Biomarker Scoring, Histological and Molecular Prediction
CONCH (Lu et al. 2024a)	2024	To develop a visual-language FM for computational pathology that overcomes limitations such as label scarcity and task-specific training.	WSIs + Text	Trained using over 1.17 million image–caption pairs sourced from various educational and clinical materials	Visual-Language pretraining using a contrastive learning approach based on the CoCa (Contrastive Captioners) framework (Yu et al. 2022)	Slide-level classification, ROI classification, cross modality retrieval
PRISM (Shaikovski et al. 2024)	2024	To efficiently aggregate information from image tiles and clinical reports to create holistic representations of WSI, combining	WSIs + Text	587,000 whole slide images (WSIs) paired with 195,000 associated clinical reports.	Adopts the CoCa framework, which comprises a vision encoder, Language Decoder (BioGPT), and Contrastive Learning.	Subtyping, biomarker prediction, clinical report generation, zero shot prediction
PLIP (Huang et al. 2023)	2023	Crowd-sourced image–text data used to train a generalizable FM.	WSIs + Text	208,414 pathology images paired with natural language descriptions.	Multimodal AI (language & image pretraining)	Slide classification, Image–Text Retrieval and Segmentation/Captioning
CTransPath (Wang et al. 2022)	2022	To develop a self-supervised feature extractor specifically designed for histopathological image classification.	WSIs	15 million tiles derived from WSIs over 25 anatomical sites and cover 32 different cancer subtypes.	Semantically Relevant Contrastive Learning (SRCL) combined with CNN backbone.	Slide and tile classification, Patch retrieval, Gland segmentation, mitosis detection
Radiology-based Foundation Models						
Foundation Model	Year	Objective/Purpose	Training Modality	Training Cohorts	Training Method	Downstream Tasks
MED-FORM (Jung et al. 2025)	2025	Medical FM that uses CT with clinical variables for multimodal cancer analysis and risk prediction	CT + Clinical Data	Pretrained on CT slicesfrom lung (141k), breast (8k), and colorectal cancer (10k)	MIL with SimCLR SSL for CT features, followed by CT–clinical alignment via contrastive learning	Cancer risk prediction, treatment response assessment and monitoring, multimodal cancer staging classification
CT-FM (Pai et al. 2025)	2025	3D image-based FM for volumetric CT understanding for clinical cancer imaging	CT	148,000 CT scans consisting of anatomical regions, cancer types, and imaging protocols	Uses label level contrastive learning for 3D volumetric data processing, stability optimization	Whole body organ segmentation, tumor segmentation and detection, cancer detection
MEDSAM2 (Ma et al. 2025)	2024	Medical segmentation model with memory attention formedical imaging and video analysis	CT + MRI + Ultrasound + multidimensional imaging	Medical dataset with over 455,000 3D image-mask pairs and 76,000 frames	Memory-attention blocks for processing sequential medical images and 3D volumes	3D organ segmentation, temporal medical image analysis, real-time ultrasound segmentation, cardiac imaging
M3FM (Niu et al. 2025)	2025	Medical multimodal FM for cancer analysis and risk prediction	CT + Clinical data	163,725 chest CT with 49 different clinical data types	Utilizes multimodal question answering with distributed parallel training along with cross modal attention mechanism.	Lung cancer risk prediction with cancer assessment across imaging and clinical modalities
CT-CLIP (Hamamci et al. 2024)	2024	Generalist FM for 3D CT	CT + Text	25,692 non-contrast 3D chest CT scans from CT-RATE, approximately 14.3 million 2D slices.	Contrastive learning approach with zero-shot multi-abnormality detection, and fine-tuning, using ClassFine and VocabFine	Multi-abnormality detection, case retrieval, multimodal interaction with CT-CHAT
CT-foundation Google (Yang et al. 2024)	2024	Converts CT volumes into rich embeddings for efficient task-specific modeling	CT + radiology report	527k CT studies with reports from 430k patients across three U.S. hospital regions	2D CoCa model (Yan et al. 2022) extended to VideoCoCa (Yu et al. 2022), trained on 500k + CT–report pairs	Hemorrhage, calcifications, lung cancer, abdominal lesions, urolithiasis, aneurysm detection
Cross-Domain Foundation Models						
Foundation Model	Year	Objective/Purpose	Training Modality	Training Cohorts	Training Method	Downstream Tasks
Biomed-Parse (Niu et al. 2025)	2025	FM for biomedical image parsing, joint object detection, and segmentation across modalities	Image + Text	6 M+ image-mask-label sets from 1 M+ images across 64 major biomedical object types	Joint pretraining using GPT-4 synthesized data from 45 existing segmentation datasets	Biomedical object segmentation, detection, recognition across 9 modalities
MedImageInsight (Codella et al. 2024)	2024	medical imaging embedding model for general domain medical imaging	Image + Text	Medical images across X-Ray, CT, MRI, dermoscopy, OCT, fundus photography, ultrasound, histopathology, mammography	Contrastive learning with medical image text pairs	Medical image classification, image search, report generation, disease classification
SeNMo (Waqas et al. 2024c)	2024	Self-Normalizing Foundation Model to address multiple omics modalities and clinical data types for cancer analysis	Multi-Omics + Clinical Data	The training of multi omics data includes gene expression, DNA methylation, miRNA expression, DNA mutations, protein expression modalities, and clinical data.	Self-normalizing approach for cross dataset generalization, multi-omics integration, domain adaptation techniques for handling institutional and technical batch effects	Overall Survival Prediction, Primary Cancer Type Classification, Tertiary Lymphoid Structures, Ratio Prediction
SAM (Huang et al. 2024b)	2024	Adapts SAM for unified segmentation across multimodal medical imaging	CT, MRI, ultrasound, X-ray, colonoscopy, WSIs, electron microscopy, fundus imaging	1,050,000 2D medical images with Approximately 6,033,000 segmentation masks	Pretrained on 11 M images with 1B masks (ViT), then fine-tuned on COS-MOS-1050 K	Organ segmentation, lesion segmentation, histopathological segmentation, everything mode evaluation
REMEDIS (Azizi et al. 2023)	2023	Generalizable FMs through combined supervised and SSL multimodal pretraining.	ImageNet-21 K, CT scans, Chest X-rays, WSIs, Mammograms, Skin Lesions, Retinal Imaging	Several data sources are used to collect multimodal data	Supervised pre-training and SSL.	Thoracic/breast cancer, tumors, segmentation, retinal and skin lesion detection

## Data Availability

No datasets were generated or analysed during the current study.
